# Contemporary trends in psychological research on conspiracy beliefs. A systematic review

**DOI:** 10.3389/fpsyg.2023.1075779

**Published:** 2023-02-08

**Authors:** Irena Pilch, Agnieszka Turska-Kawa, Paulina Wardawy, Agata Olszanecka-Marmola, Wiktoria Smołkowska-Jędo

**Affiliations:** ^1^Faculty of Social Sciences, Institute of Psychology, University of Silesia in Katowice, Katowice, Poland; ^2^Faculty of Social Sciences, Institute of Political Science, University of Silesia in Katowice, Katowice, Poland

**Keywords:** conspiracy beliefs, conspiracy thinking, conspiracy theories, conspiracies, systematic review

## Abstract

**Background:**

The number of psychological studies on conspiracy beliefs has been systematically growing for about a dozen years, but in recent years, the trend has intensified. We provided a review covering the psychological literature on conspiracy beliefs from 2018 to 2021. Halfway through this period, the COVID-19 pandemic broke out, accompanied by an explosion of movements based on conspiracy theories, intensifying researchers’ interest in this issue.

**Methods:**

Adhering to PRISMA guidelines, the review systematically searched for relevant journal articles published between 2018 and 2021. A search was done on Scopus and Web of Science (only peer-reviewed journals). A study was included if it contained primary empirical data, if specific or general conspiracy belief(s) were measured and if its correlation with at least one other psychological variable was reported. All the studies were grouped for the descriptive analysis according to the methodology used, the participants’ characteristics, the continent of origin, the sample size, and the conspiracy beliefs measurement tools. Due to substantial methodological heterogeneity of the studies, narrative synthesis was performed. The five researchers were assigned specific roles at each stage of the analysis to ensure the highest quality of the research.

**Results:**

Following the proposed methodology, 308 full-text articles were assessed for eligibility and 274 articles (417 studies) meeting the inclusion criteria were identified and included in the review. Almost half of the studies (49.6%) were conducted in European countries. The vast majority of the studies (85.7%) were carried out on samples of adult respondents. The research presents antecedents as well as (potential) consequences of conspiracy beliefs. We grouped the antecedents of conspiracy beliefs into six categories: cognitive (e.g., thinking style) motivational (e.g., uncertainty avoidance), personality (e.g., collective narcissism), psychopathology (e.g., Dark Triad traits), political (e.g., ideological orientation), and sociocultural factors (e.g., collectivism).

**Conclusion and limitations:**

The research presents evidence on the links between conspiracy beliefs and a range of attitudes and behaviors considered unfavorable from the point of view of individuals and of the society at large. It turned out that different constructs of conspiracy thinking interact with each other. The limitations of the study are discussed in the last part of the article.

## 1. Introduction

The development of research into conspiracy theories has been observed within various disciplines, including psychology. The number of psychological studies on conspiracy beliefs (CBs) has been growing systematically for about a dozen years now, but in recent years the trend has intensified. Due to the large number of such studies, conducted in different theoretical and methodological frameworks and using different measurement tools, it might be difficult to make a synthesis of the relevant literature. This was first attempted by Goreis and Voracek (2019), who published in the Frontiers in Psychology journal a systematic review of the psychological literature on CBs, covering the period from the beginning of database records until early 2018. It is also worth mentioning the review by van Mulukom et al. (2022) that summarizes 85 studies (available till March 2021) on antecedents and effects of CBs regarding COVID-19.

The current paper was planned as a continuation of the paper by Goreis and Voracek (2019) in the sense that it provides a review covering the psychological literature on CBs from a subsequent period (2018–2021). Halfway through this period, the COVID-19 pandemic broke out and it became a platform for an explosion of movements based on conspiracy theories, which in turn intensified the interest of researchers in this issue. Thus, it becomes extremely important not only to take a quantitative look at the new research, but also to look at new trends or directions of the analyses. The objective of this review was to summarize the evidence regarding antecedents and consequences of CBs. The significance of our review is based on the systematic approach that was used at all stages of the work. We hope that this paper will provide a useful resource for researchers and practitioners seeking a summary of recent psychological research on CBs.

Conspiracy theories can be defined as explanatory narratives about powerful agents collaborating secretly to achieve malevolent goals (Zonis and Joseph, 1994). The government and global corporations continue to be accused most frequently of conspiracies; however, any group perceived as influential could be charged with conspiracy (Douglas et al., 2019). There are also several terms related to conspiracy theories which should be defined. “Conspiracy beliefs” refer to beliefs in some specific conspiracy theories (Douglas et al., 2019). Specific conspiracy theories are focused on particular events or issues, e.g., the death of Princess Diana (Douglas and Sutton, 2018), the assassination of John F. Kennedy (Calfano, 2020) or 9/11 (Swami et al., 2010). Another term is “conspiracy mentality,” also referred to as “conspiracy ideation” or a tendency toward conspiracy thinking (Douglas et al., 2019). Conspiracy mentality describes the general, fundamental tendency to believe in conspiracies, which creates a monological belief system (Imhoff et al., 2022). It predicts beliefs in specific conspiracy theories—even contradictory (Wood et al., 2012) or fictitious ones (Swami et al., 2011).

Conspiracy theories are widespread in society. They constitute a part of human history but can also adapt to the present times, e.g., in terms of the forms of their dissemination (van Prooijen and Douglas, 2017). Whether we examine accounts of ancient Rome, medieval Europe, or contemporary America, conspiracy theories have inspired millions to take action. In the colonial and early Republic period, Americans feared that Catholics, Jews, Freemasons, Native Americans, and African Americans were conspiring against them. Over time, the list of potential conspirators would be extended to include bankers, rich businessmen and Mormons, and even the U.S. government (Olmstead, 2018; Uscinski, 2018). In a 2013 survey, four percent of polled Americans (12 million people) were found to believe that “shape-shifting reptilian people control our world by taking on human form and gaining political power to manipulate our societies” (Brotherton, 2015). During the 2016 presidential campaign of Donald Trump, many conspiracy theories were propagated, e.g., “Climate change is a hoax perpetrated by the Chinese” or “The pharmaceutical industry hides evidence that vaccines cause autism” (van Prooijen, 2018). According to the recent Eurobarometer data (European Commission, 2021), 28% of European citizens think that some viruses have been produced in government laboratories to diminish people’s freedom, 26% believe that cure for cancer is being hidden from people, whereas 17–18% are unsure whether these statements are true or false.

Conspiracy explanations tend to emerge especially after large-scale distressing events, such as terrorist attacks, economic crises, or epidemics (van Prooijen and Douglas, 2017). Nowadays, conspiracy theories have a greater potential to spread due to the Internet and social media (Connolly et al., 2019; Bangerter et al., 2020). However, not only external circumstances create space for conspiracy theories to spread. Firmly rooted in the literature are also studies presenting specific traits (e.g., cognitive, motivational, psychopathological) of the individual, making the latter more susceptible to conspiracy messages. The primary role of conspiracy theories covers three groups of motives: epistemic (e.g., willingness to understand and need for certainty), existential (e.g., need for security and control), and social (e.g., desire to maintain a positive image of self or in-group; Douglas et al., 2017). Conspiracy theories promise to satisfy important psychological needs and help to manage difficult situations. They make it easier to find meaning in ambiguous events and to deal with insecurity and threats (van Prooijen et al., 2020).

## 2. Methods

In this systematic review, we sought to identify the main directions and results of the latest research on CBs conducted within the framework of psychological science. We aimed to answer questions about the methodological features of the studies and also to provide a comprehensive overview of their results. We decided to prepare a systematic review with narrative synthesis rather than a meta-analysis because we sought to provide a comprehensive outline of the available research. Moreover, studies on CBs differ significantly in terms of the study designs, measures of CBs, and methods of statistical analysis. The authors often used similar construct (e.g., conspiracy mentality, conspiracy ideation). All the above factors make it very difficult to provide a synthesis of the results, even within a narrower scope. Thus, due to the substantial methodological heterogeneity of the studies, a narrative synthesis was performed.

Goreis and Voracek (2019) conducted the first systematic review devoted to psychological literature on CBs, covering the years from the beginning of database records (i.e., Scopus and Web of Science) until March 2018. Our review is intended to extend their work to cover the years 2018–2021. To do so, we adapted the search strategy and inclusion criteria of the systematic review by Goreis and Voracek (2019) in our review. A search was done on Scopus and Web of Science using the search terms “conspir* OR conspira* ideation OR conspira* belief* OR conspira* theory” and it was limited to the years 2018–2021. No limitation on language was imposed. The search was performed on 17 November 2021.

**FIGURE 1:**
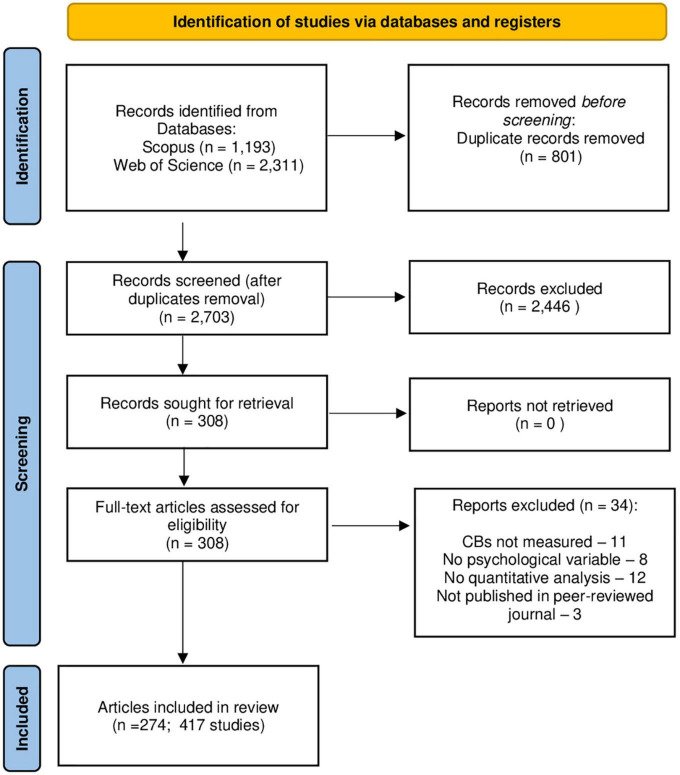
PRISMA flow diagram for the current systematic review.

Initially, 3,504 records were extracted (Web of Science = 2,311, Scopus = 1,193). After duplicates removal, we obtained 2,703 records. The screening process covered the titles and abstracts and it was performed by two researchers (IP and PW) evaluating independently and deciding whether a study met the inclusion criteria using a consensus-based screening process. A study was included if it contained primary empirical data, if specific or general conspiracy belief(s) were measured and if its correlation with at least one other psychological variable was reported. Only articles published in peer-reviewed journals were considered to ensure the quality of the studies. The language of the publication was not an exclusion criterion—one article in German (Baier and Manzoni, 2020) and one in Portuguese (Rezende et al., 2021) were included in the review. One of the articles was published both in English and Portuguese (Rezende et al., 2019a), another one in English and Spanish (Guan et al., 2021). After that, 308 full-text articles were assessed for eligibility, and 274 articles (417 studies) meeting the inclusion criteria were identified and included in the review (see Figure 1). Three reviewers (AOM, PW, and WSJ) extracted data from the studies for further analysis using a form specifically developed for this review. The other two investigators (IP and ATK) verified the data. Any disagreement was resolved by consensus.

All the studies were grouped for the descriptive analysis according to the methodology used (correlational-cross-sectional, correlational-longitudinal, experimental), participants’ characteristics (adults, school-age students, undergraduates), continent of origin, sample size, and CBs measurement tools. For the substantive analysis, the studies were grouped into two partially overlapping groups according to whether their main focus was on the antecedents or consequences of CBs. A full list of papers included in the review and a summary description of the studies are available as Supplementary material.

## 3. Results

The descriptive analysis was conducted first to give a summary of major study characteristics. Of the 417 studies described in the articles analyzed, nearly half (49.6%) were conducted in European countries (see Table 1). Other 136 (32.6%) studies were conducted in North America. The vast majority of the studies (85.7%) were carried out on a sample of adult respondents, with only 60 studies concerned undergraduates/students. Some of the studies measured specific groups of respondents, such as individuals who had not been vaccinated against COVID-19 (e.g., Yang et al., 2021) or health professionals (Al-Sanafi and Sallam, 2021). The majority of the studies (71.2%) had a cross-sectional design, and the remaining studies had an experimental design (23.3%) or a longitudinal design (5.5%).

**TABLE 1:** Characteristics of the studies included in the current review.

Characteristic	No. of studies	% of total
**Continent of origin**		
Europe	207	49.6
North America	136	32.6
Asia	28	6.7
Australia (and Oceania)	7	1.7
South America	10	2.4
Africa	1	0.2
Multiple continents	28	6.7
**Sample**		
Adults	359	85.7
Students	60	14.3
**Sample size**		
0–100	7	1.7
101–500	207	49.6
501–1,000	86	20.6
1,001–1,500	50	12.0
1,501–2,000	15	3.6
2,001–5,000	28	6.7
>5,001	24	5.8
**Study design**		
Cross-sectional	297	71.2
Experimental	97	23.3
Longitudinal	23	5.5

### 3.1. Measurement of conspiracy beliefs

Beliefs in conspiracy theories are usually measured with self-report questionnaires, referring to conspiracy mentality or specific conspiracy theories (Swami et al., 2017; Imhoff et al., 2022; see Table 2). Scales referring to specific conspiracy theories usually ask participants if they believe in a conspiratorial explanation of particular issues or events. Some examples of measures referring to specific issues include the Vaccine Conspiracy Belief Scale (VCBS; Shapiro et al., 2016) or HIV Conspiracy Theory Scale. Some measures include questions about several specific conspiracy theories which together create a general score of conspiracy ideation (Swami et al., 2017; Douglas et al., 2019), such as the Belief in Conspiracy Theories Inventory (BCTI; Swami et al., 2010).

**TABLE 2:** Questionnaires most used in the studies included in the review.

Questionnaire	Acronym	Original study	Generic form of beliefs?	Used in studies
Generic Conspiracist Beliefs Scale	GCBS	Brotherton et al., 2013	Yes	62
Conspiracy Mentality Questionnaire	CMQ	Bruder et al., 2013	Yes	57
Conspiracy Mentality Scale	CMS	Imhoff and Bruder, 2014	Yes	52
Belief in Conspiracy Theories Inventory	BCTI	Swami et al., 2010	No	17
Vaccine Conspiracy Beliefs Scale	VCBS	Shapiro et al., 2016	No	11
HIV Conspiracy Theory Scale	–	Bogart and Bird, 2003	No	5

Another type of scales measure conspiracy mentality without making reference to specific conspiracy theories (Swami et al., 2017). They consist of broader statements about conspiracies and relate to the general tendency to accept conspiracy explanations (Imhoff et al., 2022). Measures of conspiracy mentality are more stable and less skewed in distribution than measures of specific conspiracy theories. They are also more independent from other ideological content. The best-known questionnaires for the general tendency to endorse conspiracy theories are the Generic Conspiracist Beliefs Scale (GCBS; Brotherton et al., 2013) and the Conspiracy Mentality Questionnaire (CMQ; Bruder et al., 2013). The GCBS was the most frequently used measure of general belief on conspiracy theories. It was used in a total of 62 studies (see Table 2). The CMQ and the new one Conspiracy Mentality Scale (CMS; Stojanov and Halberstadt, 2019) were used in a bit fewer studies (CMQ—57, CMS—52). In turn, the BCTI, that measures endorsement of a range of conspiracy theories, was used in 17 studies.

The most commonly used scales measuring CBs were scales measuring specific conspiracy theory beliefs. Conspiratorial thinking related to COVID-19 has been studied the most. Due to the specific and new situation, there was no single scale which most researchers would use. The authors of the individual studies opted for either single-item scales (e.g., Chen et al., 2020; El-Elimat et al., 2021) or multi-item scales (e.g., Cassese et al., 2020; Heiss et al., 2021; Chayinska et al., 2022). The scale items referred most often to the origin of the virus, pointing to a specific “culprit” of the pandemic, e.g., “COVID-19 is a bacteriological weapon used by the Chinese Communist Party to create panic in the West” (Bertin et al., 2020), “Bill Gates caused (or helped cause) the spread of COVID-19 in order to expand his vaccination programs” (Agley and Xiao, 2021) or to some groups not specified in more detail, e.g., “COVID-19 is a biological weapon created by some countries to destabilize the world” (Baeza-Rivera et al., 2021). Studies also used scales related to belief in conspiracy theories about COVID-19 vaccines, creating their own scales (e.g., Cislak et al., 2021; de Sousa et al., 2021), or relying on the previous validated Vaccine Conspiracy Beliefs Scale (VCBS). In addition, medical conspiracy theories included scales related to HIV (Patev et al., 2019; Jolley et al., 2020a; Ojikutu et al., 2020; Olansky et al., 2020) and the Zika virus (Klofstad et al., 2019; Piltch-Loeb et al., 2019).

Conspiracy beliefs were also investigated with regard to members of out-groups. The relevant studies focused on specific national or religious groups: Muslims (van Prooijen et al., 2018b), Chinese people (Guan and Yang, 2020; van Prooijen and Song, 2021), and Americans (van Prooijen and Song, 2021). They were usually based on conspiracy stereotypes, with threatening out-group members being constructed as a collective enemy, aiming to take control of “us” by acting secretly. In studies embedded in political sciences, similar analyses were conducted in relation to party identification (e.g., Enders and Smallpage, 2019a) and to attitudes toward the establishment (Wood and Gray, 2019; Enders and Uscinski, 2021b).

The scales used in the studies were often specific to conspiracy theories related to political events occurring the respective country, both currently and in the past, which we illustrate by examples. In Poland, studies concerned beliefs in a conspiracy related to the Smolensk crash (“Polish and Russian authorities jointly conceal the truth about the catastrophe”; Bilewicz et al., 2019). In the UK, belief in conspiracy theories regarding Brexit was investigated (“Leave campaigner and Conservative MP Sarah Wollaston announced recently that she has changed her mind and is now backing Remain. The government have planted Remain supporters in Leave to create the appearance that Leave is losing supporters”; Jolley et al., 2022). In Pakistan, research concerned conspiracies related to four conspiracy narratives: the death of Osama bin Laden, the identity of Benazir Bhutto’s killers, the siege of the red Mosque in Islamabad, or nuclear weapons (Siddiqui, 2020). In the US, studies included those around 9/11 (“As you know, on September 11, 2001 the United States was attacked. Who do you think was behind the 9/11 attacks?”; Adam-Troian et al., 2021) or the Kennedy assassination (an experiment, exposure to media news; Calfano, 2020), and in Serbia, conspiracy theories were examined related to the war in former Yugoslavia (“The Hague Tribunal was created with the main idea to only punish the Serbs”; Milošević Đorđević et al., 2021b).

### 3.2. Links between the constructs describing conspiracy beliefs

Alongside typically used constructs (conspiracy mentality, conspiracy beliefs), researchers have also been exploring related ones, such as a Manichaean worldview, a belief in unseen forces, fatalism (Carey, 2019), a belief in an unjust world (Furnham, 2021), and dangerous world beliefs (Hart and Graether, 2018). They represent a particular manner of seeing the world and explaining the events taking place there. These general constructs of conspiratorial functioning constitute universal predispositions, not determined by sociopolitical or cultural contexts. CBs were also investigated as a part of a wider category of “unfounded beliefs” (Teličák and Halama, 2021). The generality of belief hypothesis (i.e., the generality of endorsement of various unsubstantiated claims, such as unsubstantiated conspiracy theories, scientific and psychological misconceptions, or paranormal beliefs) received support (Bensley et al., 2020). Researchers have also undertaken an analysis of the relationship between general categories of conspiratorial functioning and belief in specific conspiracy theories (Radnitz, 2022). For example, in the study by Miller (2020) a tendency toward conspiracy thinking turned out statistically significant, positive predictors of three specific CTs: “The virus is a biological weapon intentionally released by China,” “The virus was accidentally released by China,” “The virus was accidentally released by the U.S.” COVID-19 and generics CBs correlated in many studies (e.g., Georgiou et al., 2020; Alper et al., 2021; Gligorić et al., 2021; Jensen et al., 2021).

Another commonly discussed predictive factor in the belief in conspiracy theories is the tendency to believe in other conspiracy theories. Researchers also explore the relations between the belief in specific theories, verifying the proposition that one of the predictive factors in the belief of conspiracy theories is the tendency to believe in other conspiracy theories. A study conducted in Venezuela (Andrade, 2021a) looked, among other things, at theories about Simon Bolivar’s poisoning by American agents and about Chavez’s death in Havana, theories that US Military personnel brought COVID-19 to Wuhan as a biological weapon, and that COVID-19 was engineered by the Chinese government in a Wuhan lab, as a biological weapon. The study showed the proneness to believe in COVID conspiracy theories to be predicted by belief in other conspiracy theories, but only if they cohered with particular geopolitical sympathies in the context of Venezuelan politics. In the study by Miller (2020) mentioned above, the researcher investigated the correlation between belief in the individual theories indicated. According to the findings, the CTs were highly correlated and a large majority of the participants believed in more than one. Interestingly, even mutually contradictory CTs were positively related to one another.

Although different CBs were correlated in many studies, there is also evidence that the content of CBs matters. For example, general CBs and government-related conspiracies related to COVID-19 differed in their potential causes and consequences, with only the former being positive predictors of xenophobic tendencies and only the latter negatively predicting pandemic protective behavior (Oleksy et al., 2021b). Moreover, different CBs were uniquely related to the susceptibility to conjunction fallacy (Wabnegger et al., 2021). On the other hand, in the experiment by Meuer et al. (2021) not the features of conspiracy theories, but only conspiracy mentality predicted credibility judgments of different conspiracy theories.

### 3.3. Antecedents of conspiracy beliefs

Psychologists are interested in identifying diverse factors that can be viewed as potential antecedents of CBs. In this part of the review, we grouped them into six categories: cognitive, motivational, personality, psychopathology, political, and sociocultural factors. These studies seek to identify the psychological mechanisms underlying the development of CBs and point to the potential reasons of individual differences in CBs level.

#### 3.3.1. Cognitive factors

A cognitive perspective on conspiracy theories assumes that CBs can be understood as the effect of everyday cognitive processes (Douglas and Sutton, 2018). In the analyzed period, more evidence was found about the cognitive roots of CBs in both cross-sectional and experimental studies. Two main themes emerged from these studies, exploring associations between CBs and thinking skills (e.g., rational, intuitive, or critical thinking) and between CBs and cognitive biases (i.e., deviations from rational thinking). Several articles tested the relationship between intuitive and analytical thinking and CBs. Analytical and rational thinking skills measured as analytical thinking style (Ballova Mikušková, 2021; Georgiou et al., 2021a; Gligorić et al., 2021; Čavojová et al., 2022), rational thinking style (Ballová Mikušková, 2018), scientific reasoning (Georgiou et al., 2021a; Čavojová et al., 2022), critical thinking ability (Lantian et al., 2021), or cognitive reflection (Clifford et al., 2019; Rizeq et al., 2021; Pisl et al., 2021a) were negatively related to CBs. The negative association between cognitive ability (intelligence) and conspiracy mentality occurred when rationality priming was used, which suggests that interventions against CBs can be successful when they strengthen people’s motivation to be rational (Adam-Troian et al., 2019). There is also some evidence that analytical thinking is related to lower CBs only in people who value epistemic rationality (Ståhl and van Prooijen, 2018). On the other hand, an intuitive thinking style (Georgiou et al., 2019; Drinkwater et al., 2020; Pytlik et al., 2020) and faith in intuition (Alper et al., 2021) were positively related to CBs [but no relationship was found in the study by Gligorić et al. (2021)].

The associations between automatic cognitive processes and cognitive biases and CBs were also investigated. In relation to previous research suggesting that the desire to impose meaning and order was an important motive of CBs, van der Wal et al. (2018) showed that conspiracy thinking occurred when people drew implausible casual connections between co-occurring events unlikely to be directly connected. van Prooijen et al. (2018a) investigated illusory pattern perception and showed that conspiracy thinking was related to causal inferences of chaotic or random stimuli. In turn, Wagner-Egger et al. (2018) found the relationship between CBs and teleological thinking. The endorsement of conspiracy theories was also positively connected with cognitive biases: jumping to conclusion bias (Pytlik et al., 2020; Kuhn et al., 2021; Sanchez and Dunning, 2021), liberal acceptance bias, bias against disconfirmatory evidence (Georgiou et al., 2021b; Kuhn et al., 2021), possibility of being mistaken (Kuhn et al., 2021), and negatively associated with data gathering ability (Bernadyn and Feigenson, 2018) and evidence integration (Georgiou et al., 2021b). People with high and low conspiracy mentality had different reactions to cues of epistemic authoritativeness (Imhoff et al., 2018). In other studies, a tendency to accept mutually exclusive beliefs predicted specific CBs and conspiracy mentality (Petrović and Žeželj, 2021), and a meta-belief that beliefs should change according to evidence was negatively related to CBs (Pennycook et al., 2020). Interesting results were obtained in a series of experiments by Huang and Whitson (2020): mind-body dissonance/incongruence led to a compensatory control process which promoted CBs and conspiracy thinking.

#### 3.3.2. Motivational factors

A motivational perspective underlines that CBs can promise to satisfy important psychological needs. As was mentioned in the Introduction, the taxonomy proposed by Douglas et al. (2017) enables classifying these motives into three categories (epistemic, existential, or social motives). Two groups of needs were more extensively investigated: epistemic needs associated with certainty and knowledge and existential needs related to sense of personal control. The studies found positive associations of CBs with uncertainty avoidance or intolerance (Alper et al., 2021; Larsen et al., 2021; Marques et al., 2022) and need for cognitive closure (Golec de Zavala and Federico, 2018; Gligorić et al., 2021; for a different result see Boot et al., 2021). In another study, need for cognitive closure predicted a tendency toward conspiratorial explanations for uncertain events when such explanations were situationally accessible (Marchlewska et al., 2018). In a series of experiments, Kovic and Füchslin (2018) showed that conspiratorial thinking in situations when it was used as an explanation for events tended to increase as the probability of the event decreased. It was proposed that conspiratorial thinking could be viewed as a coping mechanism for uncertainty.

Need for control was another motive positively related to CBs (Gligorić et al., 2021). The compensatory control hypothesis (stating that people believe in conspiracy theories seeking compensation for their lack of control) was supported by evidence in relation to COVID-19 CBs; CBs served as a compensatory control mechanism: perceived control (associated with the COVID-19 threat) was inversely related to COVID-19 CBs, but only when other sources of compensatory control were unavailable (Stojanov et al., 2021). A negative correlation between perceived control and CBs was also reported by Mao et al. (2020), but in an experiment (Nyhan and Zeitzoff, 2018) no support was found for the hypothesis that CBs might be the result of feelings of powerlessness or lack of individual control. Nyhan and Zeitzoff (2018) quoted potential reasons for this unexpected finding (such as social desirability bias, the disproportionately young, male, and educated sample, or sincerity of respondents). In another study, motivational orientations to pursuing goals (promotion focus vs. prevention focus) were found to be related to CBs—experiments showed that promotion focus can reduce CBs because it activates a sense of personal control (Whitson et al., 2019). It is worth noting that the results described in this section do not indicate that CBs are effective in satisfying important needs. In fact, there is evidence that CBs can even strengthen feelings of existential threat (Liekefett et al., 2021). Instead, recent research showed that certain CBs can also satisfy another type of needs—CBs can be perceived by some people as entertaining and exciting, and individuals who perceived them this way were more prone to believe in conspiracy theories (van Prooijen et al., 2022b).

#### 3.3.3. Personality factors

As individuals differ in their susceptibility to CBs, some recent studies looked at relationships between CBs and personality traits and other individual-difference features. The investigations would take into account both personality factor models (Big Five, HEXACO), and temperamental traits (impulsivity, sensation seeking), evaluations of self, and trait-like constructs (such as coping styles and attachment styles). Research showed that impulsivity (Alper et al., 2021) and sensation seeking (van Prooijen et al., 2022b) were positively associated with CBs. It was also supported that narcissism and self-esteem have the opposite relationships with CBs (a positive one in the case of narcissism, and a negative one for self-esteem) and served as mutual suppressors (Siem et al., 2021). Collective narcissism measured as an individual difference was also positively related to CBs (Golec de Zavala and Federico, 2018; Marchlewska et al., 2019; Bertin et al., 2021; Stoica and Umbres̨, 2021; van Prooijen and Song, 2021; Wang et al., 2021). Further studies sought to identify relationships between CBs and general personality traits, but the results were inconsistent (in the five-factor model: positive relationships with conscientiousness and openness, Rezende et al., 2021, and in the six-factor model: negative relationships with agreeableness and conscientiousness, Bowes et al., 2021), albeit in line with the results of the meta-analysis (Goreis and Voracek, 2019) that did not find such associations. Among other individual difference features, avoidance coping (dispositional, but also situational) was associated with CBs in cross-sectional and experimental studies (Marchlewska et al., 2022), anxious attachment predicted belief in specific conspiracy theories and a general tendency (Green and Douglas, 2018) and avoidant attachment predicted conspiracy mentality (Leone et al., 2018).

#### 3.3.4. Psychopathology factors

There is extensive evidence that CBs are associated with psychopathology. Among the psychopathology factors investigated in research on CBs, subclinical forms of mental disorders (e.g., depression) and personality disorders (e.g., borderline) or their symptoms, such as paranoia, delusion proneness, dissociative tendencies, or anxiety, can be distinguished. Maladaptive, socially aversive psychological traits (referred to as the Dark Triad or the Dark Tetrad), often treated as subclinical manifestations of disorders, were also investigated. CBs correlated positively with the Dark Triad personality traits, i.e., narcissism, Machiavellianism, and psychopathy (March and Springer, 2019; Ahadzadeh et al., 2021; Bowes et al., 2021; Gligorić et al., 2021; Hughes and Machan, 2021). In another study, the positive associations between the Dark Tetrad (i.e., Dark Triad traits plus everyday sadism) sub-scales and conspiracist ideation were mediated by a tendency toward odd beliefs, fatalism, and distrust (Kay, 2021).

In the area of psychopathology, CBs were found to be positively related to paranoia (Furnham and Grover, 2021; Kuhn et al., 2021; Larsen et al., 2021; Freeman et al., 2022), schizotypy (Barron et al., 2018; Hart and Graether, 2018; Georgiou et al., 2019; Denovan et al., 2020; Dyrendal et al., 2021; Furnham and Grover, 2021), delusion proneness (Georgiou et al., 2019; Larsen et al., 2021), borderline (Furnham and Grover, 2021); psychoticism (Bowes et al., 2021; Teličák and Halama, 2021), and dissociative tendencies (Pisl et al., 2021a). The relationship between schizotypy and CBs was mediated by thinking styles (Barron et al., 2018; Denovan et al., 2020). A positive relationship of internalizing symptoms (depression, anxiety) with CBs (Sallam et al., 2020; Bowes et al., 2021; De Coninck et al., 2021) was also observed. In line with this finding, an experimental increase in COVID-19 threat evoked BIS-related emotions (such as fear and anxiety) which in turn increased CBs about the coronavirus (Jutzi et al., 2020). On the other hand, at the level of the personality disorder clusters, CBs were negatively predicted by the “anxious” cluster of personality disorders (Furnham and Grover, 2021). In another study, incidental (experimentally induced) emotions (happiness, anger, or anxiety) had no effect on the endorsement of conspiracy theories (Yu et al., 2021). The above contradictory findings suggest that the relationship between fear or anxiety and CBs can be more complex.

#### 3.3.5. Political factors

Political factors stand out from among the others due to their area of reference, namely the broadly perceived political space. They shape citizens’ activity within the political system (including their interactions with political actors). They comprise both political attitudes (e.g., populism) and the factors shaping them (e.g., political powerlessness), as well as mechanisms of political functioning of individuals (e.g., political ideology). The largest amount of space in this area was dedicated to ideological orientation (Federico et al., 2018; Golec de Zavala and Federico, 2018; Hart and Graether, 2018; Hollander, 2018; Vitriol and Marsh, 2018; Enders and Smallpage, 2019b; Featherstone et al., 2019; Calvillo et al., 2020; Agley and Xiao, 2021; Enders and Uscinski, 2021a; Furnham, 2021; Min, 2021; Nera et al., 2021; Stecula and Pickup, 2021; Stoica and Umbres̨, 2021; Tonković et al., 2021; Stojanov and Douglas, 2022), with extremist ideology distinguished in some studies (Federico et al., 2018; Golec de Zavala and Federico, 2018; Baier and Manzoni, 2020; Enders and Uscinski, 2021a; van der Linden et al., 2021; Walter and Drochon, 2022). Party identification was included in several studies (Hollander, 2018; Vitriol and Marsh, 2018; Enders and Smallpage, 2019a,b; Enders and Uscinski, 2021a; Stecula and Pickup, 2021). The results of the research are not consistent, which may be related, among other things, to the political culture of the specific country. However, a tendency can be observed toward stronger associations of the extreme poles of the identification scales with CBs. Extremist thinking, whether left- or right-wing, as an unambiguous style of defining the world, based on concrete axioms, gives meaning to social and political events more easily. Rottweiler and Gill (2022) found that a stronger conspiracy mentality led to increased violent extremist intentions. However, this relationship is contingent on several individual differences, such as lower self-control, holding a weaker law-relevant morality, and scoring higher in self-efficacy.

A consistent direction of positive relationships is demonstrated by CBs and authoritarianism (Federico et al., 2018; Golec de Zavala and Federico, 2018; Enders and Smallpage, 2019b; Stojanov et al., 2019; Wood and Gray, 2019; Baier and Manzoni, 2020; Goldberg and Richey, 2020; Dyrendal et al., 2021; Kim and Kim, 2021; Krüppel et al., 2021; Milošević Đorđević et al., 2021a; Tonković et al., 2021). Right-wing authoritarianism as a political stance characterized by obedience to an authoritarian leader, and a belief in a hierarchical social order may in fact function as a defense system to protect the socio-political *status quo*.

Research shows positive relations between populist attitudes and CBs (Cargnino, 2021; Eberl et al., 2021). This relationship, however, turned out to be more complicated in a Chinese study by Guan and Yang (2020), who identified two subtypes of populism (right- vs. responsibility-oriented) and two subtypes of conspiracy beliefs (pro-system vs. anti-system). The results demonstrated that while right-oriented populism was positively correlated with anti-system CBs, it had no significant correlations with pro-system CBs. Responsibility-driven populism was positively correlated with pro-system CBs, and negatively correlated with anti-system CBs. Against this background, it is interesting to look at the study by Jolley et al. (2018), showing that conspiracy theories, often presented as alternatives to the narrative of the establishment, might strengthen rather than undermine support for the social *status quo*, if the latter’s legitimacy is threatened.

Other political variables investigated in the CBs context include political knowledge (Golec de Zavala and Federico, 2018; Gemenis, 2021; Min, 2021), political cynicism (Vitriol and Marsh, 2018; Milošević Đorđević et al., 2021a), political deprivation (Baier and Manzoni, 2020), political powerlessness (Tonković et al., 2021), anomie (Baier and Manzoni, 2020; Majima and Nakamura, 2020), ostracism (Poon et al., 2020), corruption perception (Milošević Đorđević et al., 2021a), and political interest (Mondak, 2020). The results generally showed that people who feel alienated within the social and political system, do not find the strength to act politically, or perceive the political system as inaccessible for the average citizen present a higher level of CBs. It has also been proven that inclusion partisan stimuli significantly decrease CBs for supporters of one party and increase such beliefs for supporters of the other party (Enders and Smallpage, 2018).

#### 3.3.6. Sociocultural factors

This section presents social and cultural factors that predict susceptibility to conspiracy theories. Relationships between CBs and values are reported first, following by studies exploring predictors of CBs associated with communication process and media use, social trust and religion. This section ends with the description of findings that do not fit within the above categories.

A series of studies showed relationships between CBs and Hofstede’s cultural values (measured on both the national and individual levels)—positive for collectivism and masculinity, regardless of the measure of CBs used (Adam-Troian et al., 2021). Rezende et al. (2019b) reported correlations between CBs and excitement, suprapersonal, interactive, and promotion values (from the Basic Values Survey). Binding moral foundations (but not individualizing moral foundations) were positively associated with CBs (Leone et al., 2019). There is also evidence from a study conducted in the USA and China that cultural dimension promoting hierarchy in society (i.e., power distance) is related to increased intergroup CBs (van Prooijen and Song, 2021).

De Coninck et al. (2021) obtained associations between main sources of information and the inclination to believe in conspiracies about the coronavirus (traditional media use, health experts—negative associations, digital media use, politicians, personal contacts—positive associations). In another study, the relationships between social media use and different CBs were conditional on the predisposition to conspiracy thinking (stronger for those with higher levels of conspiracy thinking; Enders et al., 2021). In turn, social media skepticism was a negative predictor of CBs about COVID-19 (Ahadzadeh et al., 2021). Pro-conspiracy messages increased CBs regardless of the form of such messages (explicit or implicit conspiracy cues), but subsequently receiving corrective information had the opposite effect on CBs (Bolsen and Druckman, 2018; Lyons et al., 2019). On the other hand, in an experiment by Nera et al. (2018) the impact of narratives on CBs was not observed. In another study priming resistance to persuasion decreased CBs (Bonetto et al., 2018). Brotherton and Son (2021) discovered that claims regarding conspiracies were situated by participants between facts and opinions, and the extent to which such claims were perceived as facts was associated with the degree to which the individual agreed or disagreed with them.

Another variable introduced in many models during the analyzed period was trust, examined in different subject contexts. One of the more commonly used constructs was institutional trust. It was measured most often by the general trust in institutions (Jasinskaja-Lahti and Jetten, 2019; Baier and Manzoni, 2020; Eberl et al., 2021; Milošević Đorđević et al., 2021a; Šrol et al., 2021; Stojanov and Douglas, 2022), but in some studies the institutions were specifically identified, e.g., the parliament (Vezzoni et al., 2022), the World Health Organization (Freeman et al., 2022), the United Nations, the European Union (Freeman et al., 2022), the government (Freeman et al., 2022; Kim and Kim, 2021), heath institutions (Bruder and Kunert, 2022), public officials (Walter and Drochon, 2022), or media (Stojanov and Douglas, 2022). In the pandemic situation, the scientific community undertook immediate research ensuring smooth access to medical and social studies on a huge scale. In the social space, including particularly the virtual space, peer-reviewed scientific research functioned alongside emerging content bearing the hallmarks of misinformation or conspiratorial narratives. Thus, trust in science, scientists and research naturally emerged among the correlates of CBs (Fasce and Picó, 2019; Agley and Xiao, 2021; Constantinou et al., 2021b; Eberl et al., 2021; Jensen et al., 2021; Stecula and Pickup, 2021; Tonković et al., 2021; Bruder and Kunert, 2022; Freeman et al., 2022). The studies also came to include some classic measures: social trust (Golec de Zavala and Federico, 2018; Nestik et al., 2020; Kim and Kim, 2021), or interpersonal trust (Hollander, 2018; Vitriol and Marsh, 2018). In the vast majority of the cases, the results obtained yielded negative relationships between CBs and trust. In a small number of cases, these relationships were statistically insignificant (e.g., Vitriol and Marsh, 2018; Kim and Kim, 2021).

An important place in the area of social factors is occupied by analyses of the relationship between CBs and religiousness. Researchers see a similarity between an all-powerful being (as described in many religions) and a hidden power organizing events or hiding the truth. This undefinable power is a fundamental feature of conspiracy thinking (Galliford and Furnham, 2017). Although in some studies religious individuals were more likely than non-religious ones to believe in conspiracy theories (Kim and Kim, 2021; Leibovitz et al., 2021; Tonković et al., 2021; Freeman et al., 2022), other studies found no significant relationship (e.g., Agley and Xiao, 2021; Andrade, 2021c; Furnham, 2021; Teličák and Halama, 2021), or the relationship was different for different CBs scales (Atari et al., 2019). In a study by Jasinskaja-Lahti and Jetten (2019), no differences were found between endorsement of CBs between believers and non-believers. These discrepancies show how difficult it is to conceptualize and operationalize the construct of religiousness as such. In fact, the analyses presented different approaches, classifying religiousness for instance in terms of religious commitment (Agley and Xiao, 2021), religious belief (Freeman et al., 2022), religion (Furnham, 2021), religiosity (Hart and Graether, 2018; Kim and Kim, 2021), the importance of religion (Tonković et al., 2021), or spirituality (Gligorić et al., 2021; Kosarkova et al., 2021). The nature of traditionally understood religiosity is institutional, but nowadays more and more people have more popular and unorganized spiritual beliefs (Baker and Draper, 2010; Yilmaz, 2021). It is worth pointing out that researchers outline the relations between religiosity and spirituality differently in their studies of CBs. Teličák and Halama (2021) approach the two constructs autonomously, recording weak positive relations between CBs and spirituality and religiosity (slightly stronger for spirituality). Kosarkova et al. (2021) demonstrated an interesting relationship, namely that spirituality without being religiously affiliated was linked to high levels of vaccination refusal and hesitancy, whereas affiliation to a church showed no significant associations. In the study by Gligorić et al. (2021), spirituality emerged as the most significant predictor of higher conspiracy endorsement. Some researchers treat religion and spirituality jointly without drawing differences between the constructs (Leibovitz et al., 2021; Marques et al., 2022), obtaining positive correlations with CBs.

A series of studies showed a tendency to overestimate the CBs of others; perceived CBs of in-groups (but not out-groups) predicted strongly personal CBs, which suggests that challenging misperceived conspiracy belief norms might be the way to reduce CBs (Cookson et al., 2021). There is also evidence that CBs emerging as a response to victimizing social events can destroy social cohesion (Bilewicz et al., 2019) and that chronic social devaluation gives rise to African American endorsement of race-relevant CBs (Davis et al., 2018).

### 3.4. Consequences of conspiracy beliefs

The endorsement of conspiracy theories may have a range of negative consequences for both individuals and the society at large. Results of studies dedicated to this aspect are presented below, first research focusing on the implications CBs have for individual health and wellbeing, followed by studies describing the implications of CBs important from the point of view of social wellbeing. Further on in this section, the relations between CBs and political and health-related attitudes directly linked to the COVID-19 pandemic will be discussed.

Negative emotions occupy an important position among the potential negative implications of CBs considered from the point of view of an individual endorsing such beliefs. Belief in COVID-19 conspiracies predicted higher fear, distress, and anxiety (Chen et al., 2020; Jolley et al., 2020b; Jovančević and Milićević, 2020; Leibovitz et al., 2021), future anxiety (Duplaga and Grysztar, 2021), and also lower wellbeing (Spasovski and Kenig, 2020; van Prooijen et al., 2021; Freeman et al., 2022) and life satisfaction (Kohút et al., 2022). These implications are also visible in the working environment in the form of a negative impact of CBs on job search behavior (Gabriel et al., 2021) and lower job satisfaction (Chen et al., 2020).

From the point of view of social wellbeing, what seems important are the relations between CBs and various unfavorable attitudes, such as anti-science attitudes (Marques et al., 2022), climate skepticism (Hornsey et al., 2018a), lower prosocial orientation (Hornsey et al., 2021), resistance to humanitarian aid (Mashuri et al., 2022), pharmacophobia (Petelinšek and Lauri Korajlija, 2020), negative attitudes toward HIV testing (Patev et al., 2019; Hood et al., 2020), and other socially unfavorable attitudes (Jedinger, 2021; Molz and Stiller, 2021). The implications of CBs potentially affecting social communication include: endorsement of fake news (Anthony and Moulding, 2019; Halpern et al., 2019; Faragó et al., 2020; Frischlich et al., 2021), rating nonsense as profound (Čavojová et al., 2019), and a willingness to share conspiracy theories online (Lobato et al., 2020). In turn, when it comes to popularization of scientific knowledge, CBs were related to believing in viral and deceptive claims about science (Landrum and Olshansky, 2019), perceiving pseudo-scientific arguments as stronger (Landrum et al., 2021), and a tendency to reject complex scientific messages and to feel rejected and devalued reading such messages (Schnepf et al., 2021).

CBs can be also connected with phenomena constituting manifestations of serious social pathologies, such as social stigma and fear of social exclusion (Lantian et al., 2018), dehumanization of others (Markowitz et al., 2021), criminal intentions and support for violence (Jolley et al., 2019; Jolley and Paterson, 2020). The potential negative social implications of CBs are also suggested by the relations between HIV/AIDS CBs and a lower intention to adopt pre-exposure prophylaxis as HIV prevention (Brooks et al., 2018; Jolley et al., 2020a; Parent et al., 2020) and the relationship between CBs and an increase in preferences for alternative therapies over biomedical ones (Lamberty and Imhoff, 2018). It is worth adding that even short-term exposure to conspiracy theories can affect actual behavior (Bolsen et al., 2020; Balafoutas et al., 2021; Meuer and Imhoff, 2021).

A vast majority of the studies covered by this review were conducted in the period of the COVID-19 pandemic. It was a time when researchers would seek the factors determining conventional and unconventional attitudes and behaviors of citizens with regard to decisions of the authorities (e.g., adherence to guidelines aiming to reduce the spread of COVID-19), as well as the attitudes and behaviors toward political actors (e.g., voting behavior). Similarly, a lot of space in the literature was devoted to the search for predictors of health-promoting behaviors. Both lines of research, partly overlapping, dominated the investigations into the implications of CBs, because political and health-related consequences of CBs seemed especially important in the times of the COVID-19 pandemic.

Several studies investigated the role of CBs in shaping political behavior; in particular, strong connections are visible between CBs and anti-government activity aimed at changing the existing order. Seeing the world as governed by conspiracies increased the intentions to engage in illegal political actions (such as participation in illegal demonstrations or committing a violent attack) but attenuated the willingness to engage in legal forms of political participation (e.g., voting or joining a political party; Imhoff et al., 2021). Moreover, belief in conspiracy theories makes it possible to anticipate unconventional (but non-violent) participation (Ardèvol-Abreu et al., 2020), justification of protest actions (Chayinska and Minescu, 2018), support for leaving the EU (Jolley et al., 2021), support for Brexit (Swami et al., 2018), foreign policy views (Onderco and Stoeckel, 2020), and Stealth Democracy beliefs (Pantazi et al., 2021). CBs were also associated with self-reported voting behavior in the 2016 Italian constitutional referendum (Mancosu et al., 2021) and voting behavior with regard to the election of Donald Trump (Lamberty et al., 2018). CBs were positively related to political activities such as talking to people about voting for or against a candidate or a party, or signing a petition on paper about a political or social issue (Kim, 2022).

Social and political consequences of health decisions of individuals seem especially important in the times of the COVID-19 pandemic. Therefore, it is not unexpected that researchers have most recently been focusing on identifying the antecedents of pandemic-related health behaviors. Conspiracy theory endorsement turned out to be one of the frequently included predictors of such behavior. There is evidence that health-related CBs can lower health-seeking intentions (Natoli and Marques, 2021). In many studies, anti-COVID-19 health protective attitudes and behavior were negatively related to COVID-19 CBs (Biddlestone et al., 2020; Bierwiaczonek et al., 2020; Earnshaw et al., 2020; Egorova et al., 2020; Kowalski et al., 2020; Rieger, 2020; Romer and Jamieson, 2020; Abadi et al., 2021; Allington et al., 2021; Farias and Pilati, 2021; Karić and Međedović, 2021; Resnicow et al., 2021; Soveri et al., 2021; Chayinska et al., 2022; Latkin et al., 2022; Pavela Banai et al., 2022; Garry et al., 2022; Hughes et al., 2022; Pummerer et al., 2022) and conspiracy mentality (Gualda et al., 2021; Oleksy et al., 2021a; Pellegrini et al., 2021; Plohl and Musil, 2021; Maftei and Holman, 2022). However, sometimes no relationship (Prichard and Christman, 2020; Alper et al., 2021; Naveed et al., 2021; Schnell et al., 2021; Šuriņa et al., 2021; Yarosh et al., 2021) or even positive relationships (Alper et al., 2021; Corbu et al., 2021; Wang and Kim, 2021) between these variables were reported. This may be the case because different CBs about COVID-19 can have different and opposite behavioral consequences depending on the content of the conspiracies (Imhoff and Lamberty, 2020; Chan et al., 2021; Jia and Luo, 2021). Moreover, people with high conspiracy mentality can engage in non-normative pseudoscientific preventive behavior (Marinthe et al., 2020; Teovanović et al., 2021). The specificity of preventive behavior can also differentiate the relationship between CBs and behavior (Bruder and Kunert, 2022). In other studies, COVID-19 CBs were used as mediators (Maglić et al., 2021; Swami and Barron, 2021) and conspiracy mentality was used as a moderator (Lazarević et al., 2021) of the relationships between other predictors and preventive behavior. In turn, psychological flexibility (Constantinou et al., 2021a), institutional trust and self-perceived infections (van Prooijen et al., 2022a) served as mediators between CBs and health behavior.

A lot of studies conducted in the reviewed period evaluated the importance of different factors in predicting attitudes and behaviors associated with vaccination. This is understandable considering the importance of vaccination in the context of COVID-19 pandemic. CBs were frequently included as predictors in these studies. The majority of studies concerned COVID-19 vaccines and provided strong evidence for negative relationships between COVID-related CBs and the COVID-19 vaccine attitudes and the intention to be vaccinated (Bertin et al., 2020; Goldberg and Richey, 2020; Al-Sanafi and Sallam, 2021; Al-Wutayd et al., 2021; Andrade, 2021a,b; Arshad et al., 2021; Burke et al., 2021; de Sousa et al., 2021; Eberhardt and Ling, 2021; El-Elimat et al., 2021; Jensen et al., 2021; Kachurka et al., 2021; Lindholt et al., 2021; Martinez-Berman et al., 2021; Pisl et al., 2021a; Pivetti et al., 2021a; Ruiz and Bell, 2021; Sallam et al., 2021a,b; Sowa et al., 2021; Wirawan et al., 2021; Woolf et al., 2021). General CBs were also negative predictors of COVID-19 vaccination attitudes (Rozbroj et al., 2019; Bertin et al., 2020; Andrade, 2021a; Jennings et al., 2021; Pisl et al., 2021a,b; Sallam et al., 2021a; Bacon and Taylor, 2022; Knobel et al., 2022; Nazlı et al., 2022). However, some studies did not find such a relationship (Baeza-Rivera et al., 2021; Guillon and Kergall, 2021; Yang et al., 2021). Other studies established the mediational role of COVID-19 CBs (Maftei and Holman, 2021, 2022; Simione et al., 2021), or conspiracy mentality (Scrima et al., 2022) between other predictors and the intention to be vaccinated. Similar relationships were obtained for non-COVID vaccines (Hornsey et al., 2018b,^2020^, ^2021^; Callaghan et al., 2019; Fonseca et al., 2021; Milošević Đorđević et al., 2021a; Pivetti et al., 2021a,b).

The vast majority of the studies on the relationship between CBs and health attitudes and behavior were correlational. However, the results of several experimental studies are also available. For example, Chen et al. (2021) used the theory of planned behavior to create an experiment. After exposure to HPV vaccine-related conspiracy messages, participants presented more negative attitudes toward the vaccine and weaker intentions to receive the vaccine (Chen et al., 2021). Experimental investigation of the effectiveness of different methods of reducing the acceptance of COVID-related CBs showed that the science- and fact-focusing corrections were effective (Guan et al., 2021). In another study, transparent negative communication about the COVID-19 vaccine decreased acceptance of the vaccine but also increased trust in health authorities, whereas vague, reassuring communication lowered trust and boosted CBs but did not increase vaccine acceptance (Petersen et al., 2021).

## 4. Discussion

The objective of the current review was to provide an extensive overview of the empirical studies on CBs within psychology. We present a synthesis of the results of 274 articles published between 2018 and 2021 identified in accordance with the guidelines for systematic reviews. It should be underlined that about half of the respective period coincided with the pandemic period, posing an extraordinary challenge for individuals and institutions, and also resulting in a great number of new conspiracy theories. The current paper presents antecedents as well as consequences of CBs. We grouped the potential antecedents of CBs into six categories: cognitive, motivational, personality, psychopathology, political, and sociocultural factors. Within cognitive psychology, researchers have explored basic cognitive processes, such as illusory pattern perception, and different cognitive biases that can lead to CBs. Growing evidence suggests that analytical thinking is associated with a lower tendency to believe in conspiracies. Within the motivational perspective, relationships were demonstrated between conspiracy thinking and important needs and motives, such as uncertainty avoidance, need for cognitive closure, or need for control. Among individual differences, pathological traits and disorders (such as schizotypy, paranoia, and depression) have gained more attention of conspiracy theory researchers than normal personality traits. The Dark Triad personality traits were also often investigated in the context of CBs.

Among the political antecedents of CBs, researchers analyzed political attitudes and mechanisms of political functioning of individuals. Ideological orientation and party identification were included most often in the research models. Although the research results are not consistent, a clear association can be seen between extremist views and CBs. Analyses of other variables such as anomie, political deprivation and political powerlessness show that poorly perceived political subjectivity predisposes one more strongly to CBs. Although in the analyzed period, relations were sought mainly between CBs and right-wing authoritarianism, it should be emphasized that more recent literature reveals certain paths aimed at analyzing the relations between CBs and left-wing authoritarianism features (Avendaño et al., 2022; Costello et al., 2022). Left-wing authoritarianism predicts higher endorsement of vaccines and support for compulsory vaccination against COVID-19 and penalties for unvaccinated people (Peng, 2022). Galais and Guinjoan (2022) show that people that value security over freedom are more prone to falling for pandemic misbeliefs. CBs are associated with a belief in a hierarchical social order (right-wing) and with anti-hierarchical attitudes about social order (left-wing). In the sociocultural factors group, the researchers looked for links between CBs and cultural values. Relationships between conspiratorial thinking and social media use and the perception of various media content were also confirmed. Research shows that low trust is more strongly associated with conspiratorial thinking. In turn, the associations between religiousness and CBs did not always yield consistent results.

New trends in research into CBs will be identified by comparing the studies covered by this review to the results of the systematic review by Goreis and Voracek (2019), covering studies from 1994 until early 2018. This is made possible by applying identical criteria for selecting the studies covered by these reviews. The one by Goreis and Voracek (2019) included seven papers, also included in this review, since the original and final year of their publication differed. These papers were treated (only for the purpose of comparing the two sets of papers) as components of the set of the systematic review by Goreis and Voracek, and at the same time they were excluded from the set of papers covered by this review to avoid their double attribution.

**FIGURE 2:**
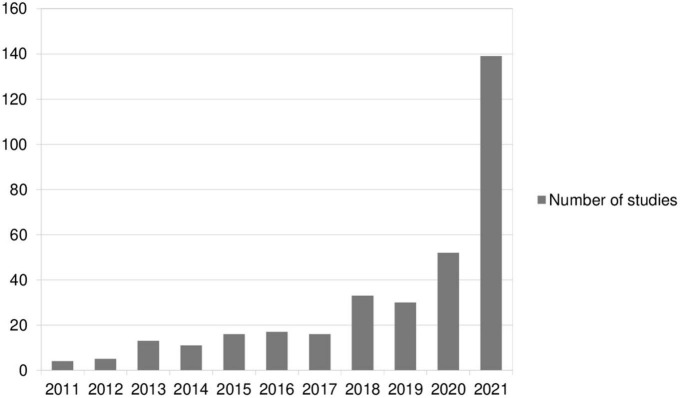
Number of studies on conspiracy beliefs per year based on Goreis and Voracek (2019) and the current review.

The comparison between the number of papers included in comparable literature reviews (96 vs. 267, after removing duplicates) shows the dramatic increase in the number of studies published in the years 2018–2021. One of the factors that could be responsible for the increase the interest of researchers in the topic of CBs is the COVID-19 pandemic outbreak that resulted in the emergence of a number of conspiracy theories (related to the origin of the virus, the process of its spread, the consequences or composition of vaccines, etc.) spreading rapidly around the world. The pandemic was a difficult situation, generating many doubts, difficult emotions and, above all, a lack of prospects for many individuals. Thus, this period saw a boom in conspiracy theories providing quick answers to difficult questions. Because of their widespread and universal nature (presence in different cultures), as well as of their easy-to-grasp effect on attitudes and behaviors, it has been easier to conduct comparative studies across cultures, including populations hitherto underrepresented in research of this type. Older research on CBs was conducted mainly on WEIRD (White, Educated, Industrialized, Reach, and Democratic) samples, which hinders the generalization of their results. The number of studies included in both reviews, grouped by publication year, is shown in Figure 2.

In the period covered by the present review, an identical share of cross-sectional studies (71.2%) was recorded compared to the previous period (Goreis and Voracek, 2019; 71,1%). However, the pandemic situation in course encouraged the researchers to perform longitudinal studies (5.5%), not present earlier. The territorial scope of the research was expanded. While the majority of studies continue to be performed in Europe and North America, more studies were recorded in Asia (increase from 4.2 to 6.7%), and some first studies appeared in Africa (nine studies, including multiple continents research). An increase in the number of studies involving more numerous samples was recorded. The number of small studies conducted on fewer than 100 people decreased by far (from 16.3 to 1.7%), while the percentage of research on the most numerous samples of over 500 people increased (from 19.9 to 49.6%). A decrease was recorded in the number of studies on students. In the period covered by our review, 2.5 times fewer studies on students (14.3%) were carried out compared to the previous period in total on the group of graduate and undergraduate students (36.8%). This may have been related to the more difficult access to that group at a time when classes had been suspended in most countries, or were being held online.

The pandemic situation became a source for yet another important trend, namely the increase in the number of practically-oriented studies. In the original review by Goreis and Voracek (2019), few such studies appeared. They concerned, among other things, diagnosing CBs among future teachers with an analysis of the benefits of critical thinking courses as a way of reducing conspiracy beliefs (Ballová Mikušková, 2018) and health-harming behaviors as consequences of CBs (Jolley and Douglas, 2014; Oliver and Wood, 2014). The post-2020 situation saw the emergence of a line of research that involved seeking the factors potentially supporting citizens’ behaviors oriented toward containment of virus spread. In that line of research, CBs constitutes, among other things, a predictor of negative attitudes toward vaccines (e.g., Burke et al., 2021; de Sousa et al., 2021; Eberhardt and Ling, 2021) or boycotting the authorities, or unconventional activity such as participating in demonstrations and protests (e.g., Chayinska and Minescu, 2018; Ardèvol-Abreu et al., 2020).

Goreis and Voracek (2019) pointed out in their systematic review that the majority of research on CBs published until 2018 lacked theoretical background. This conclusion seems to be valid also for the studies included in this review. A similar opinion was expressed by van Prooijen and Douglas (2018, p. 898) in the Introduction to European Journal of Social Psychology Special Issue on conspiracy theories. They stated that “the field is lacking a solid theoretical framework that contextualizes previous findings, that enables novel predictions, and that suggests interventions to reduce the prevalence of conspiracy theories in society.” In the reviewed period, such a theoretical framework, accepted by researchers exploring various themes empirically in the field of research into CBs, does not seem to have appeared. However, over the past few years, a number of papers have been published with the aim of summarizing the current knowledge from the psychological point of view and of outlining the direction of further research (Douglas et al., 2017; Douglas and Sutton, 2018; van Prooijen and Douglas, 2018; van Prooijen and van Vugt, 2018; van Prooijen, 2020; Biddlestone et al., 2021). For example, van Prooijen and Douglas (2018) defined four basic principles of CBs (i.e., the consequential, universal, emotional, and social character of such beliefs), drawn from empirical studies. van Prooijen and van Vugt (2018) proposed an evolutionary model of CBs. van Prooijen (2020) put forward the existential threat model of CBs, asserting that experiencing existential threat triggers epistemic sense-making processes which in turn can lead to CBs only when antagonistic groups are salient. In turn, Biddlestone et al. (2021) presented a model in which CBs are motivated by the frustration of motives associated with three selves (individual, relational, and collective).

Douglas et al. (2017) articulated the need for research on the consequences of CBs. During the period under examination, a significant increase was observed in the number of studies focusing on this topic. However, it is worth noting that most of the studies on potential consequences of CBs had cross-sectional designs and thus causal relationships remained unclear. In the times of the COVID-19 pandemic, when health-related behaviors had an especially high impact on both individual lives and social security and welfare, most of the research on the effects of CBs focused on this particular issue. These studies provided strong evidence that specific COVID-19 beliefs and conspiracy mentality can predict adherence to pandemic measures and a broad range of pandemic-related attitudes and behaviors, including attitudes toward vaccination. Several studies investigated the role of CBs in shaping political behavior. CBs made it possible to predict activity aimed at changing the existing socio-political order for instance through demonstrations or illegal political actions.

The vast majority of research emphasizes the negative individual and social consequences of endorsing CBs. However, positive effects for individuals (such as satisfying their needs) are also potentially possible. van Prooijen (2022) listed the potential psychological benefits connected with a conspiracy worldview as ego-defensive benefits, help in rationalizing the individual’s behavior, and entertainment. Despite the skepticism often expressed by researchers regarding the possibility of satisfying needs as a result of endorsing conspiracy theories, further research is needed to resolve this problem (see: Liekefett et al., 2021).

## 5. Limitations

The analyses were restricted to studies published between 2018 and 2021, which is a relatively short period of time. However, during that time a rapid increase in the number of studies on CBs was observed, which was the motivation for this review. Only published papers retrieved from two databases (Scopus and Web of Science) were used in the review. These indexing databases seem to be the most appropriate considering the theme of the review, and they are widely regarded as high quality sources of scientific articles, but this decision reduces the number of sources taken into account. Thus, it is likely that not all important, relevant studies were included in our review. The rationale behind this decision was that we strived to maintain compliance with the solutions applied by Goreis and Voracek (2019) in their systematic review (see the Current study section). It is also worth noting that our intention was to present the state of knowledge on CBs as widely as possible at this point in time in order to organize and inspire conspiracy theory researchers rather than formulate answers regarding more specific issues (such as prevalence, evaluation of interventions, or measurement issues).

Although validated and reliable measures of CBs were used in many reviewed studies, some of them used very short (1- to 3-item) scales prepared for the particular research, which makes the comparison of the results very difficult. Study publication and outcome reporting biases can affect the results of systematic reviews, especially when meta-analyses were conducted. However, in this study, due to the broadly formulated purpose of the review and the diversity of the reviewed studies, statistical synthesis was not performed. There is also a risk that some errors were made during data extraction. To avoid this, two independent reviewers (using a consensus-based method) were engaged at every stage of preparing the review.

## Data availability statement

The original contributions presented in this study are included in this article/Supplementary material, further inquiries can be directed to the corresponding author.

## Author contributions

IP and AT-K: conception and design and interpretation of the results. IP and PW: systematic literature search. WS-J, AO-M, PW, AT-K, and IP: analysis of the results. IP, AT-K, PW, AO-M, and WS-J: compilation of the results. All authors approved the submitted version for publication.

## Supplementary material

Supplementary Table 1 Summary description of the studies.

Supplementary Appendix 1 Bibliographic data of the papers included in the review.
