# Supplementary material for: Contemporary trends in psychological research on conspiracy beliefs. A systematic review
Source: Front Psychol. 2023 Feb 8;14:1075779. doi: 10.3389/fpsyg.2023.1075779 (PMC9945548; doi:10.3389/fpsyg.2023.1075779)
Supplement: Supplementary Appendix 1 — Bibliographic data of the papers included in the review. [file Data_Sheet_1.docx]

**Research papers included in the systematic review:**

1. Abadi, D., Arnaldo, I., & Fischer, A. (2021). Anxious and Angry: Emotional Responses to the COVID-19 Threat. *Frontiers in Psychology*, *12*. https://www.frontiersin.org/article/10.3389/fpsyg.2021.676116
2. Adam-Troian, J., Caroti, D., Arciszewski, T., & Ståhl, T. (2019). Unfounded beliefs among teachers: The interactive role of rationality priming and cognitive ability. *Applied Cognitive Psychology*, *33*(4), 720–727. https://doi.org/10.1002/acp.3547
3. Adam-Troian, J., Wagner-Egger, P., Motyl, M., Arciszewski, T., Imhoff, R., Zimmer, F., Klein, O., Babinska, M., Bangerter, A., Bilewicz, M., Blanuša, N., Bovan, K., Bužarovska, R., Cichocka, A., Çelebi, E., Delouvée, S., Douglas, K. M., Dyrendal, A., Gjoneska, B., … van Prooijen, J.-W. (2021). Investigating the Links Between Cultural Values and Belief in Conspiracy Theories: The Key Roles of Collectivism and Masculinity. *Political Psychology*, *42*(4), 597–618. https://doi.org/10.1111/pops.12716
4. Agley, J., & Xiao, Y. (2021). Misinformation about COVID-19: Evidence for differential latent profiles and a strong association with trust in science. *BMC Public Health*, *21*(1), 89. https://doi.org/10.1186/s12889-020-10103-x
5. Ahadzadeh, A. S., Ong, F. S., & Wu, S. L. (2021). Social media skepticism and belief in conspiracy theories about COVID-19: The moderating role of the dark triad. *Current Psychology*. https://doi.org/10.1007/s12144-021-02198-1
6. Allington, D., Duffy, B., Wessely, S., Dhavan, N., & Rubin, J. (2021). Health-protective behaviour, social media usage and conspiracy belief during the COVID-19 public health emergency. *Psychological Medicine*, *51*(10), 1763–1769. https://doi.org/10.1017/S003329172000224X
7. Alper, S., Bayrak, F., & Yilmaz, O. (2021). Psychological correlates of COVID-19 conspiracy beliefs and preventive measures: Evidence from Turkey. *Current Psychology*, *40*(11), 5708–5717. https://doi.org/10.1007/s12144-020-00903-0
8. Al-Sanafi, M., & Sallam, M. (2021). Psychological Determinants of COVID-19 Vaccine Acceptance among Healthcare Workers in Kuwait: A Cross-Sectional Study Using the 5C and Vaccine Conspiracy Beliefs Scales. *Vaccines*, *9*(7), 701. https://doi.org/10.3390/vaccines9070701
9. Al-Wutayd, O., Khalil, R., & Rajar, A. B. (2021). Sociodemographic and Behavioral Predictors of COVID-19 Vaccine Hesitancy in Pakistan. *Journal of Multidisciplinary Healthcare*, *14*, 2847–2856. https://doi.org/10.2147/JMDH.S325529
10. Andrade, G. (2021a). Belief in Conspiracy Theories About COVID-19 Amongst Venezuelan Students: A Pilot Study. *Revista Colombiana de Psicología*, *30*(1), 79–88. https://doi.org/10.15446/rcp.v30n1.87357
11. Andrade, G. (2021b). Covid-19 vaccine hesitancy, conspiracist beliefs, paranoid ideation and perceived ethnic discrimination in a sample of University students in Venezuela. *Vaccine*, *39*(47), 6837–6842. https://doi.org/10.1016/j.vaccine.2021.10.037
12. Andrade, G. (2021c). Vaccine hesitancy and religiosity in a sample of university students in Venezuela. *Human Vaccines & Immunotherapeutics*, *17*(12), 5162–5167. https://doi.org/10.1080/21645515.2021.1981737
13. Anthony, A., & Moulding, R. (2019). Breaking the news: Belief in fake news and conspiracist beliefs. *Australian Journal of Psychology*, *71*(2), 154–162. https://doi.org/10.1111/ajpy.12233
14. Ardèvol-Abreu, A., Gil de Zúñiga, H., & Gámez, E. (2020). The influence of conspiracy beliefs on conventional and unconventional forms of political participation: The mediating role of political efficacy. *British Journal of Social Psychology*, *59*(2), 549–569.
15. Arshad, M. S., Hussain, I., Mahmood, T., Hayat, K., Majeed, A., Imran, I., Saeed, H., Iqbal, M. O., Uzair, M., Rehman, A. ur, Ashraf, W., Usman, A., Syed, S. K., Akbar, M., Chaudhry, M. O., Ramzan, B., Islam, M., Saleem, M. U., Shakeel, W., … Rasool, M. F. (2021). A National Survey to Assess the COVID-19 Vaccine-Related Conspiracy Beliefs, Acceptability, Preference, and Willingness to Pay among the General Population of Pakistan. *Vaccines*, *9*(7), 720. https://doi.org/10.3390/vaccines9070720
16. Atari, M., Afhami, R., & Swami, V. (2019). Psychometric assessments of Persian translations of three measures of conspiracist beliefs. PloS One, 14(4), e0215202.
17. Bacon, A. M., & Taylor, S. (2021). Vaccination Hesitancy and Conspiracy Beliefs in the UK During the SARS-COV-2 (COVID-19) Pandemic. *International Journal of Behavioral Medicine*. https://doi.org/10.1007/s12529-021-10029-7
18. Baeza-Rivera, M. J., Salazar-Fernández, C., Araneda-Leal, L., & Manríquez-Robles, D. (2021). To get vaccinated or not? Social psychological factors associated with vaccination intent for COVID-19. *Journal of Pacific Rim Psychology*, *15*, 18344909211051800. https://doi.org/10.1177/18344909211051799
19. Baier, D., & Manzoni, P. (2020). Verschwörungsmentalität und Extremismus – Befunde aus Befragungsstudien in der Schweiz (Conspiracy mentality and extremism – Survey findings from Switzerland). *Monatsschrift fur Kriminologie und Strafrechtsreform*, *103*(2), 83–96. Scopus. https://doi.org/10.1515/mks-2020-2044
20. Balafoutas, L., Libman, A., Selamis, V., & Vollan, B. (2021). Exposure to conspiracy theories in the lab. *Economic and Political Studies*, *9*(1), 90–112. https://doi.org/10.1080/20954816.2020.1818930
21. Ballová Mikušková, E. (2018). Conspiracy Beliefs of Future Teachers. *Current Psychology*, *37*(3), 692–701. https://doi.org/10.1007/s12144-017-9561-4
22. Barron, D., Furnham, A., Weis, L., Morgan, K. D., Towell, T., & Swami, V. (2018). The relationship between schizotypal facets and conspiracist beliefs via cognitive processes. *Psychiatry Research*, *259*, 15–20. https://doi.org/10.1016/j.psychres.2017.10.001
23. Bensley, D. A., Lilienfeld, S. O., Rowan, K. A., Masciocchi, C. M., & Grain, F. (2020). The generality of belief in unsubstantiated claims. *Applied Cognitive Psychology*, *34*(1), 16–28. https://doi.org/10.1002/acp.3581
24. Bernadyn, T., & Feigenson, K. A. (2018). Data gathering ability contributes to visual organization and probabilistic reasoning. *Heliyon*, *4*(3), e00582. https://doi.org/10.1016/j.heliyon.2018.e00582
25. Bertin, P., Nera, K., & Delouvée, S. (2020). Conspiracy Beliefs, Rejection of Vaccination, and Support for hydroxychloroquine: A Conceptual Replication-Extension in the COVID-19 Pandemic Context. *Frontiers in Psychology*, *11*. https://www.frontiersin.org/article/10.3389/fpsyg.2020.565128
26. Bertin, P., Nera, K., Hamer, K., Uhl-Haedicke, I., & Delouvée, S. (2021). Stand out of my sunlight: The mediating role of climate change conspiracy beliefs in the relationship between national collective narcissism and acceptance of climate science. *Group Processes & Intergroup Relations*, *24*(5), 738–758. https://doi.org/10.1177/1368430221992114
27. Biddlestone, M., Green, R., & Douglas, K. M. (2020). Cultural orientation, power, belief in conspiracy theories, and intentions to reduce the spread of COVID-19. *British Journal of Social Psychology*, *59*(3), 663–673. https://doi.org/10.1111/bjso.12397
28. Bierwiaczonek, K., Kunst, J. R., & Pich, O. (2020). Belief in COVID-19 Conspiracy Theories Reduces Social Distancing over Time. *Applied Psychology: Health and Well-Being*, *12*(4), 1270–1285. https://doi.org/10.1111/aphw.12223
29. Bilewicz, M., Witkowska, M., Pantazi, M., Gkinopoulos, T., & Klein, O. (2019). Traumatic rift: How conspiracy beliefs undermine cohesion after societal trauma? *Europe’s Journal of Psychology*, *15*(1), 82.
30. Bolsen, T., & Druckman, J. N. (2018). Validating Conspiracy Beliefs and Effectively Communicating Scientific Consensus. *Weather, Climate, and Society*, *10*(3), 453–458. https://doi.org/10.1175/WCAS-D-17-0096.1
31. Bolsen, T., Palm, R., & Kingsland, J. T. (2020). Framing the Origins of COVID-19. *Science Communication*, *42*(5), 562–585. https://doi.org/10.1177/1075547020953603
32. Bonetto, E., Troïan, J., Varet, F., Lo Monaco, G., & Girandola, F. (2018). Priming Resistance to Persuasion decreases adherence to Conspiracy Theories. *Social Influence*, *13*(3), 125–136. https://doi.org/10.1080/15534510.2018.1471415
33. Boot, A. B., Eerland, A., Jongerling, J., Verkoeijen, P. P., & Zwaan, R. A. (2021). Gathering, processing, and interpreting information about COVID-19. *Scientific Reports*, *11*(1), 1–17.
34. Bowes, S. M., Costello, T. H., Ma, W., & Lilienfeld, S. O. (2021). Looking under the tinfoil hat: Clarifying the personological and psychopathological correlates of conspiracy beliefs. *Journal of Personality*, *89*(3), 422–436. https://doi.org/10.1111/jopy.12588
35. Brooks, R. A., Allen, V. C., Regan, R., Mutchler, M. G., Cervantes-Tadeo, R., & Lee, S.-J. (2018). HIV/AIDS conspiracy beliefs and intention to adopt preexposure prophylaxis among black men who have sex with men in Los Angeles. *International Journal of STD & AIDS*, *29*(4), 375–381. https://doi.org/10.1177/0956462417727691
36. Brotherton, R., & Son, L. K. (2021). Metacognitive Labeling of Contentious Claims: Facts, Opinions, and Conspiracy Theories. *Frontiers in Psychology*, *12*, 924.
37. Bruder, M., & Kunert, L. (2022). The conspiracy hoax? Testing key hypotheses about the correlates of generic beliefs in conspiracy theories during the COVID-19 pandemic. *International Journal of Psychology*, *57*(1), 43–48. https://doi.org/10.1002/ijop.12769
38. Burke, P. F., Masters, D., & Massey, G. (2021). Enablers and barriers to COVID-19 vaccine uptake: An international study of perceptions and intentions. *Vaccine*, *39*(36), 5116–5128. https://doi.org/10.1016/j.vaccine.2021.07.056
39. Calfano, B. R. (2020). Government-Corroborated Conspiracies: Motivating Response to (and Belief in) a Coordinated Crime. *PS: Political Science & Politics*, *53*(1), 64–71.
40. Callaghan, T., Motta, M., Sylvester, S., Lunz Trujillo, K., & Blackburn, C. C. (2019). Parent psychology and the decision to delay childhood vaccination. *Social Science & Medicine*, *238*, 112407. https://doi.org/10.1016/j.socscimed.2019.112407
41. Calvillo, D. P., Ross, B. J., Garcia, R. J. B., Smelter, T. J., & Rutchick, A. M. (2020). Political Ideology Predicts Perceptions of the Threat of COVID-19 (and Susceptibility to Fake News About It). *Social Psychological and Personality Science*, *11*(8), 1119–1128. https://doi.org/10.1177/1948550620940539
42. Carey, J. M. (2019). Who believes in conspiracy theories in Venezuela? *Latin American Research Review*, *54*(2), 444–457.
43. Cargnino, M. (2021). The Interplay of Online Network Homogeneity, Populist Attitudes, and Conspiratorial Beliefs: Empirical Evidence From a Survey on German Facebook Users. *International Journal of Public Opinion Research*, *33*(2), 337–353. https://doi.org/10.1093/ijpor/edaa036
44. Cassese, E. C., Farhart, C. E., & Miller, J. M. (2020). Gender Differences in COVID-19 Conspiracy Theory Beliefs. *Politics & Gender*, *16*(4), 1009–1018. https://doi.org/10.1017/S1743923X20000409
45. Čavojová, V., Secară, E.-C., Jurkovič, M., & Šrol, J. (2019). Reception and willingness to share pseudo-profound bullshit and their relation to other epistemically suspect beliefs and cognitive ability in Slovakia and Romania. *Applied Cognitive Psychology*, *33*(2), 299–311. https://doi.org/10.1002/acp.3486
46. Čavojová, V., Šrol, J., & Ballová Mikušková, E. (2022). How scientific reasoning correlates with health-related beliefs and behaviors during the COVID-19 pandemic? *Journal of Health Psychology*, *27*(3), 534–547. https://doi.org/10.1177/1359105320962266
47. Chan, H.-W., Chiu, C. P.-Y., Zuo, S., Wang, X., Liu, L., & Hong, Y. (2021). Not-so-straightforward links between believing in COVID-19-related conspiracy theories and engaging in disease-preventive behaviours. *Humanities and Social Sciences Communications*, *8*(1), 1–10. https://doi.org/10.1057/s41599-021-00781-2
48. Chayinska, M., & Minescu, A. (2018). “They’ve conspired against us”: Understanding the role of social identification and conspiracy beliefs in justification of ingroup collective behavior. *European Journal of Social Psychology*, *48*(7), 990–998. https://doi.org/10.1002/ejsp.2511
49. Chayinska, M., Uluğ, Ö. M., Ayanian, A. H., Gratzel, J. C., Brik, T., Kende, A., & McGarty, C. (2021). Coronavirus conspiracy beliefs and distrust of science predict risky public health behaviours through optimistically biased risk perceptions in Ukraine, Turkey, and Germany. *Group Processes & Intergroup Relations*, 1368430220978278. https://doi.org/10.1177/1368430220978278
50. Chen, L., Zhang, Y., Young, R., Wu, X., & Zhu, G. (2021). Effects of Vaccine-Related Conspiracy Theories on Chinese Young Adults’ Perceptions of the HPV Vaccine: An Experimental Study. *Health Communication*, *36*(11), 1343–1353. https://doi.org/10.1080/10410236.2020.1751384
51. Chen, X., Zhang, S. X., Jahanshahi, A. A., Alvarez-Risco, A., Dai, H., Li, J., & Ibarra, V. G. (2020). Belief in a COVID-19 conspiracy theory as a predictor of mental health and well-being of health care workers in Ecuador: Cross-sectional survey study. *JMIR Public Health and Surveillance*, *6*(3), e20737.
52. Cislak, A., Marchlewska, M., Wojcik, A. D., Śliwiński, K., Molenda, Z., Szczepańska, D., & Cichocka, A. (2021). National narcissism and support for voluntary vaccination policy: The mediating role of vaccination conspiracy beliefs. *Group Processes and Intergroup Relations*, *24*(5), 701–719. Scopus. https://doi.org/10.1177/1368430220959451
53. Clifford, S., Kim, Y., & Sullivan, B. W. (2019). An improved question format for measuring conspiracy beliefs. *Public Opinion Quarterly,* 83(4), 690-722.
54. Constantinou, M., Gloster, A. T., & Karekla, M. (2021). I won’t comply because it is a hoax: Conspiracy beliefs, lockdown compliance, and the importance of psychological flexibility. *Journal of Contextual Behavioral Science*, *20*, 46–51. https://doi.org/10.1016/j.jcbs.2021.03.001
55. Constantinou, M., Kagialis, A., & Karekla, M. (2021). COVID-19 Scientific Facts vs. Conspiracy Theories: Is Science Failing to Pass Its Message? *International Journal of Environmental Research and Public Health*, *18*(12), 6343. https://doi.org/10.3390/ijerph18126343
56. Cookson, D., Jolley, D., Dempsey, R. C., & Povey, R. (2021). “If they believe, then so shall I”: Perceived beliefs of the in-group predict conspiracy theory belief. *Group Processes and Intergroup Relations*, *24*(5), 759–782. Scopus. https://doi.org/10.1177/1368430221993907
57. Corbu, N., Negrea-Busuioc, E., Udrea, G., & Radu, L. (2021). Romanians’ willingness to comply with restrictive measures during the COVID-19 pandemic: Evidence from an online survey. *Journal of Applied Communication Research*, *49*(4), 369–386. https://doi.org/10.1080/00909882.2021.1912378
58. Davis, J., Wetherell, G., & Henry, P. J. (2018). Social devaluation of African Americans and race-related conspiracy theories. *European Journal of Social Psychology*, *48*(7), 999–1010. Scopus. https://doi.org/10.1002/ejsp.2531
59. De Coninck, D., d’Haenens, L., & Matthijs, K. (2020). Perceived vulnerability to disease and attitudes towards public health measures: COVID-19 in Flanders, Belgium. *Personality and Individual Differences*, *166*, 110220. https://doi.org/10.1016/j.paid.2020.110220
60. de Sousa, Á. F. L., Teixeira, J. R. B., Lua, I., de Oliveira Souza, F., Ferreira, A. J. F., Schneider, G., de Carvalho, H. E. F., de Oliveira, L. B., Lima, S. V. M. A., de Sousa, A. R., de Araújo, T. M. E., Camargo, E. L. S., Oriá, M. O. B., Craveiro, I., de Araújo, T. M., Mendes, I. A. C., Ventura, C. A. A., Sousa, I., de Oliveira, R. M., … Fronteira, I. (2021). Determinants of COVID-19 Vaccine Hesitancy in Portuguese-Speaking Countries: A Structural Equations Modeling Approach. *Vaccines*, *9*(10), 1167. https://doi.org/10.3390/vaccines9101167
61. Denovan, A., Dagnall, N., Drinkwater, K., Parker, A., & Neave, N. (2020). Conspiracist beliefs, intuitive thinking, and schizotypal facets: A further evaluation. *Applied Cognitive Psychology*, *34*(6), 1394–1405. https://doi.org/10.1002/acp.3716
62. Drinkwater, K. G., Dagnall, N., Denovan, A., & Neave, N. (2020). Psychometric assessment of the generic conspiracist beliefs scale. *Plos One*, 15(3), e0230365.
63. Duplaga, M. (2020). The determinants of conspiracy beliefs related to the COVID-19 pandemic in a nationally representative sample of internet users. *International Journal of Environmental Research and Public Health*, *17*(21), 7818.
64. Duplaga, M., & Grysztar, M. (2021). The Association between Future Anxiety, Health Literacy and the Perception of the COVID-19 Pandemic: A Cross-Sectional Study. *Healthcare*, *9*(1), 43. https://doi.org/10.3390/healthcare9010043
65. Dyrendal, A., Kennair, L. E. O., & Bendixen, M. (2021). Predictors of belief in conspiracy theory: The role of individual differences in schizotypal traits, paranormal beliefs, social dominance orientation, right wing authoritarianism and conspiracy mentality. *Personality and Individual Differences*, *173*, 110645. https://doi.org/10.1016/j.paid.2021.110645
66. Earnshaw, V. A., Eaton, L. A., Kalichman, S. C., Brousseau, N. M., Hill, E. C., & Fox, A. B. (2020). COVID-19 conspiracy beliefs, health behaviors, and policy support. *Translational Behavioral Medicine*, *10*(4), 850–856. https://doi.org/10.1093/tbm/ibaa090
67. Eberhardt, J., & Ling, J. (2021). Predicting COVID-19 vaccination intention using protection motivation theory and conspiracy beliefs. *Vaccine*, *39*(42), 6269–6275. https://doi.org/10.1016/j.vaccine.2021.09.010
68. Eberl, J.-M., Huber, R. A., & Greussing, E. (2021). From populism to the “plandemic”: Why populists believe in COVID-19 conspiracies. *Journal of Elections, Public Opinion and Parties*, *31*(sup1), 272-284.
69. Egorova, M. s, Parshikova, O., Chertkova, Y., Staroverov, V., & Mitina, O. (2020). COVID-19: Belief in Conspiracy Theories and the Need for Quarantine. *Psychology in Russia: State of the Art*, *13*, 3–25. https://doi.org/10.11621/pir.2020.0401
70. El-Elimat, T., AbuAlSamen, M. M., Almomani, B. A., Al-Sawalha, N. A., & Alali, F. Q. (2021). Acceptance and attitudes toward COVID-19 vaccines: A cross-sectional study from Jordan. *PLOS ONE*, *16*(4), e0250555. https://doi.org/10.1371/journal.pone.0250555
71. Enders, A. M., & Smallpage, S. M. (2018). On the measurement of conspiracy beliefs. *Research & Politics,* 5(1), 2053168018763596.
72. Enders, A. M., & Smallpage, S. M. (2019a). Informational cues, partisan-motivated reasoning, and the manipulation of conspiracy beliefs. *Political Communication*, *36*(1), 83–102.
73. Enders, A. M., & Smallpage, S. M. (2019b). Who are conspiracy theorists? A comprehensive approach to explaining conspiracy beliefs. *Social Science Quarterly*, *100*(6), 2017–2032.
74. Enders, A. M., & Uscinski, J. E. (2021). Are misinformation, antiscientific claims, and conspiracy theories for political extremists? *Group Processes & Intergroup Relations*, *24*(4), 583–605.
75. Enders, A. M., Uscinski, J. E., Seelig, M. I., Klofstad, C. A., Wuchty, S., Funchion, J. R., Murthi, M. N., Premaratne, K., & Stoler, J. (2021). The Relationship Between Social Media Use and Beliefs in Conspiracy Theories and Misinformation. *Political Behavior*. https://doi.org/10.1007/s11109-021-09734-6
76. Faragó, L., Kende, A., & Krekó, P. (2020). We Only Believe in News That We Doctored Ourselves. *Social Psychology*, *51*(2), 77–90. https://doi.org/10.1027/1864-9335/a000391
77. Farias, J., & Pilati, R. (2021). COVID-19 as an undesirable political issue: Conspiracy beliefs and intolerance of uncertainty predict adhesion to prevention measures. *Current Psychology*, 1–11.
78. Fasce, A., & Picó, A. (2019). Science as a Vaccine. *Science & Education*, *28*(1), 109–125. https://doi.org/10.1007/s11191-018-00022-0
79. Featherstone, J. D., Bell, R. A., & Ruiz, J. B. (2019). Relationship of people’s sources of health information and political ideology with acceptance of conspiratorial beliefs about vaccines. *Vaccine*, *37*(23), 2993–2997. https://doi.org/10.1016/j.vaccine.2019.04.063
80. Federico, C. M., Williams, A. L., & Vitriol, J. A. (2018). The role of system identity threat in conspiracy theory endorsement. *European Journal of Social Psychology*, *48*(7), 927–938. https://doi.org/10.1002/ejsp.2495
81. Fonseca, I. C., Pereira, A. I., & Barros, L. (2021). Portuguese parental beliefs and attitudes towards vaccination. *Health Psychology and Behavioral Medicine*, *9*(1), 422–435. https://doi.org/10.1080/21642850.2021.1920948
82. Freeman, D., Waite, F., Rosebrock, L., Petit, A., Causier, C., East, A., Jenner, L., Teale, A.-L., Carr, L., Mulhall, S., Bold, E., & Lambe, S. (2022). Coronavirus conspiracy beliefs, mistrust, and compliance with government guidelines in England. *Psychological Medicine*, *52*(2), 251–263. https://doi.org/10.1017/S0033291720001890
83. Frischlich, L., Hellmann, J. H., Brinkschulte, F., Becker, M., & Back, M. D. (2021). Right-wing authoritarianism, conspiracy mentality, and susceptibility to distorted alternative news. *Social Influence*, *16*(1), 24–64. https://doi.org/10.1080/15534510.2021.1966499
84. Furnham, A. (2021). Just world beliefs, personal success and beliefs in conspiracy theories. *Current Psychology*. https://doi.org/10.1007/s12144-021-01576-z
85. Furnham, A., & Grover, S. (2021). Do you have to be mad to believe in conspiracy theories? Personality disorders and conspiracy theories. *International Journal of Social Psychiatry*, 00207640211031614. https://doi.org/10.1177/00207640211031614
86. Gabriel, A. S., MacGowan, R. L., Ganster, M. L., & Slaughter, J. E. (2021). The influence of COVID-induced job search anxiety and conspiracy beliefs on job search effort: A within-person investigation. *Journal of Applied Psychology*, *106*(5), 657–673. https://doi.org/10.1037/apl0000926
87. Garry, J., Ford, R., & Johns, R. (2020). Coronavirus conspiracy beliefs, mistrust, and compliance: taking measurement seriously. *Psychological Medicine*, 1-11.
88. Gemenis, K. (2021). Explaining Conspiracy Beliefs and Scepticism around the COVID-19 Pandemic. *Swiss Political Science Review*, *27*(2), 229–242.
89. Georgiou, N., Delfabbro, P., & Balzan, R. (2019). Conspiracy beliefs in the general population: The importance of psychopathology, cognitive style and educational attainment. *Personality and Individual Differences*, *151*, 109521. https://doi.org/10.1016/j.paid.2019.109521
90. Georgiou, N., Delfabbro, P., & Balzan, R. (2020). COVID-19-related conspiracy beliefs and their relationship with perceived stress and pre-existing conspiracy beliefs. *Personality and Individual Differences*, *166*, 110201. https://doi.org/10.1016/j.paid.2020.110201
91. Georgiou, N., Delfabbro, P., & Balzan, R. (2021a). Conspiracy theory beliefs, scientific reasoning and the analytical thinking paradox. *Applied Cognitive Psychology*, *35*(6), 1523–1534. https://doi.org/10.1002/acp.3885
92. Georgiou, N., Delfabbro, P., & Balzan, R. (2021b). Conspiracy-Beliefs and Receptivity to Disconfirmatory Information: A Study Using the BADE Task. *SAGE Open*, *11*(1), 21582440211006132. https://doi.org/10.1177/21582440211006131
93. Gligorić, V., da Silva, M. M., Eker, S., van Hoek, N., Nieuwenhuijzen, E., Popova, U., & Zeighami, G. (2021). The usual suspects: How psychological motives and thinking styles predict the endorsement of well-known and COVID-19 conspiracy beliefs. *Applied Cognitive Psychology*, *35*(5), 1171–1181. https://doi.org/10.1002/acp.3844
94. Goldberg, Z. J., & Richey, S. (2020). Anti-vaccination beliefs and unrelated conspiracy theories. *World Affairs,* 183(2), 105-124.
95. Golec de Zavala, A., & Federico, C. M. (2018). Collective narcissism and the growth of conspiracy thinking over the course of the 2016 United States presidential election: A longitudinal analysis. *European Journal of Social Psychology*, *48*(7), 1011–1018. https://doi.org/10.1002/ejsp.2496
96. Green, R., & Douglas, K. M. (2018). Anxious attachment and belief in conspiracy theories. *Personality and Individual Differences*, *125*, 30–37. https://doi.org/10.1016/j.paid.2017.12.023
97. Gualda, E., Krouwel, A., Palacios-Gálvez, M., Morales-Marente, E., Rodríguez-Pascual, I., & García-Navarro, E. B. (2021). Social distancing and COVID-19: Factors associated with compliance with social distancing norms in Spain. *Frontiers in Psychology*, 12.
98. Guan, T., Liu, T., & Yuan, R. (2021). Combatiendo la desinformación: Cinco métodos para contrarrestar las teorías de conspiración en la pandemia de Covid-19 (Facing disinformation: Five methods to counter conspiracy theories amid the Covid-19 pandemic). *Comunicar*, *29*(69), 71–83.
99. Guan, T., & Yang, Y. (2020). Diversifying Conspiracy Beliefs and Populist Ideologies in the Chinese Context. *Social Science Quarterly*, *101*(2), 459–472. https://doi.org/10.1111/ssqu.12766
100. Guillon, M., & Kergall, P. (2021). Factors associated with COVID-19 vaccination intentions and attitudes in France. *Public Health*, *198*, 200–207. https://doi.org/10.1016/j.puhe.2021.07.035
101. Hart, J., & Graether, M. (2018). Something’s Going on Here. *Journal of Individual Differences*, *39*(4), 229–237. https://doi.org/10.1027/1614-0001/a000268
102. Heiss, R., Gell, S., Röthlingshöfer, E., & Zoller, C. (2021). How threat perceptions relate to learning and conspiracy beliefs about COVID-19: Evidence from a panel study. *Personality and Individual Differences*, *175*. Scopus. https://doi.org/10.1016/j.paid.2021.110672
103. Hollander, B. A. (2018). Partisanship, individual differences, and news media exposure as predictors of conspiracy beliefs. *Journalism & Mass Communication Quarterly*, *95*(3), 691–713.
104. Hood, K. B., Hall, C. J., Owens, B. D., Patev, A. J., & Belgrave, F. Z. (2020). HIV Testing Behaviors among Black Rural Women: The Moderating Role of Conspiracy Beliefs and Partner Status Disclosure. *Ethnicity & Disease*, *30*(2), 251–260. https://doi.org/10.18865/ed.30.2.251
105. Hornsey, M. J., Chapman, C. M., Alvarez, B., Bentley, S., Salvador Casara, B. G., Crimston, C. R., Ionescu, O., Krug, H., Preya Selvanathan, H., Steffens, N. K., & Jetten, J. (2021). To what extent are conspiracy theorists concerned for self versus others? A COVID-19 test case. *European Journal of Social Psychology*, *51*(2), 285–293. https://doi.org/10.1002/ejsp.2737
106. Hornsey, M. J., Finlayson, M., Chatwood, G., & Begeny, C. T. (2020). Donald Trump and vaccination: The effect of political identity, conspiracist ideation and presidential tweets on vaccine hesitancy. *Journal of Experimental Social Psychology*, *88*, 103947. https://doi.org/10.1016/j.jesp.2019.103947
107. Hornsey, M. J., Harris, E. A., & Fielding, K. S. (2018a). The psychological roots of anti-vaccination attitudes: A 24-nation investigation. *Health Psychology*, *37*(4), 307–315. https://doi.org/10.1037/hea0000586
108. Hornsey, M. J., Harris, E. A., & Fielding, K. S. (2018b). Relationships among conspiratorial beliefs, conservatism and climate scepticism across nations. *Nature Climate Change*, *8*(7), 614–620. https://doi.org/10.1038/s41558-018-0157-2
109. Huang, L., & Whitson, J. (2020). Organizational costs of compensating for mind-body dissonance through conspiracies and superstitions. *Organizational Behavior and Human Decision Processes*, *156*, 1–12. https://doi.org/10.1016/j.obhdp.2019.09.006
110. Hughes, J. P., Efstratiou, A., Komer, S. R., Baxter, L. A., Vasiljevic, M., & Leite, A. C. (2022). The impact of risk perceptions and belief in conspiracy theories on COVID-19 pandemic-related behaviours. *PLOS ONE*, *17*(2), e0263716. https://doi.org/10.1371/journal.pone.0263716
111. Hughes, S., & Machan, L. (2021). It’s a conspiracy: Covid-19 conspiracies link to psychopathy, Machiavellianism and collective narcissism. *Personality and Individual Differences*, *171*, 110559. https://doi.org/10.1016/j.paid.2020.110559
112. Imhoff, R., Dieterle, L., & Lamberty, P. (2021). Resolving the Puzzle of Conspiracy Worldview and Political Activism: Belief in Secret Plots Decreases Normative but Increases Nonnormative Political Engagement. *Social Psychological and Personality Science*, *12*(1), 71–79. https://doi.org/10.1177/1948550619896491
113. Imhoff, R., & Lamberty, P. (2020). A Bioweapon or a Hoax? The Link Between Distinct Conspiracy Beliefs About the Coronavirus Disease (COVID-19) Outbreak and Pandemic Behavior. *Social Psychological and Personality Science*, *11*(8), 1110–1118. https://doi.org/10.1177/1948550620934692
114. Imhoff, R., Lamberty, P., & Klein, O. (2018). Using Power as a Negative Cue: How Conspiracy Mentality Affects Epistemic Trust in Sources of Historical Knowledge. *Personality and Social Psychology Bulletin*, *44*(9), 1364–1379. https://doi.org/10.1177/0146167218768779
115. Jasinskaja-Lahti, I., & Jetten, J. (2019). Unpacking the relationship between religiosity and conspiracy beliefs in Australia. *British Journal of Social Psychology*, *58*(4), 938–954. https://doi.org/10.1111/bjso.12314
116. Jedinger, A. (2021). Conspiracy Mentality Predicts Public Opposition to Foreign Trade. *Frontiers in Psychology*, *12*. https://www.frontiersin.org/article/10.3389/fpsyg.2021.658919
117. Jennings, W., Stoker, G., Bunting, H., Valgarðsson, V. O., Gaskell, J., Devine, D., McKay, L., & Mills, M. C. (2021). Lack of Trust, Conspiracy Beliefs, and Social Media Use Predict COVID-19 Vaccine Hesitancy. *Vaccines*, *9*(6), 593. https://doi.org/10.3390/vaccines9060593
118. Jensen, E. A., Pfleger, A., Herbig, L., Wagoner, B., Lorenz, L., & Watzlawik, M. (2021). What Drives Belief in Vaccination Conspiracy Theories in Germany? *Frontiers in Communication*, *6*. https://www.frontiersin.org/article/10.3389/fcomm.2021.678335
119. Jia, H., & Luo, X. (2021). I Wear a Mask for My Country: Conspiracy Theories, Nationalism, and Intention to Adopt Covid-19 Prevention Behaviors at the Later Stage of Pandemic Control in China. *Health Communication*, *0*(0), 1–9. https://doi.org/10.1080/10410236.2021.1958982
120. Jolley, D., Douglas, K. M., Leite, A. C., & Schrader, T. (2019). Belief in conspiracy theories and intentions to engage in everyday crime. *British Journal of Social Psychology*, *58*(3), 534–549. https://doi.org/10.1111/bjso.12311
121. Jolley, D., Douglas, K. M., Marchlewska, M., Cichocka, A., & Sutton, R. M. (2022). Examining the links between conspiracy beliefs and the EU “Brexit” referendum vote in the UK: Evidence from a two-wave survey. *Journal of Applied Social Psychology*, *52*(1), 30–36. https://doi.org/10.1111/jasp.12829
122. Jolley, D., Douglas, K. M., & Sutton, R. M. (2018). Blaming a Few Bad Apples to Save a Threatened Barrel: The System-Justifying Function of Conspiracy Theories. *Political Psychology*, *39*(2), 465–478. https://doi.org/10.1111/pops.12404
123. Jolley, D., Jaspal, R., Jolley, D., & Jaspal, R. (2020). Discrimination, HIV conspiracy theories and pre-exposure prophylaxis acceptability in gay men. *Sexual Health*, *17*(6), 525–533. https://doi.org/10.1071/SH20154
124. Jolley, D., & Paterson, J. L. (2020). Pylons ablaze: Examining the role of 5G COVID-19 conspiracy beliefs and support for violence. *British Journal of Social Psychology*, *59*(3), 628–640.
125. Jolley, D., Douglas, K. M., Skipper, Y., Thomas, E., & Cookson, D. (2021). Measuring adolescents’ beliefs in conspiracy theories: Development and validation of the Adolescent Conspiracy Beliefs Questionnaire (ACBQ). *British Journal of Developmental Psychology*, 39(3), 499-520.
126. Jovančević, A., & Milićević, N. (2020). Optimism-pessimism, conspiracy theories and general trust as factors contributing to COVID-19 related behavior – A cross-cultural study. *Personality and Individual Differences*, *167*, 110216. https://doi.org/10.1016/j.paid.2020.110216G
127. Jutzi, C. A., Willardt, R., Schmid, P. C., & Jonas, E. (2020). Between conspiracy beliefs, ingroup bias, and system justification: How people use defense strategies to cope with the threat of COVID-19. *Frontiers in Psychology*, 2538.
128. Kachurka, R., Krawczyk, M., & Rachubik, J. (2021). Persuasive messages will not increase COVID-19 vaccine acceptance: evidence from a nationwide online experiment. *Vaccines,* 9(10), 1113.
129. Karić, T., & Međedović, J. (2021). Covid-19 conspiracy beliefs and containment-related behaviour: The role of political trust. *Personality and Individual Differences*, *175*, 110697. https://doi.org/10.1016/j.paid.2021.110697
130. Kay, C. S. (2021). Actors of the most fiendish character: Explaining the associations between the Dark Tetrad and conspiracist ideation. *Personality and Individual Differences*, *171*, 110543. https://doi.org/10.1016/j.paid.2020.110543
131. Kim, S., & Kim, S. (2021). Searching for General Model of Conspiracy Theories and Its Implication for Public Health Policy: Analysis of the Impacts of Political, Psychological, Structural Factors on Conspiracy Beliefs about the COVID-19 Pandemic. *International Journal of Environmental Research and Public Health*, *18*(1), 266. https://doi.org/10.3390/ijerph18010266
132. Kim, Y. (2022). How conspiracy theories can stimulate political engagement. *Journal of Elections, Public Opinion and Parties*, *32*(1), 1–21. https://doi.org/10.1080/17457289.2019.1651321
133. Klofstad, C. A., Uscinski, J. E., Connolly, J. M., & West, J. P. (2019). What drives people to believe in Zika conspiracy theories? *Palgrave Communications*, *5*(1), 1–8. https://doi.org/10.1057/s41599-019-0243-8
134. Knobel, P., Zhao, X., & White, K. M. (2022). Do conspiracy theory and mistrust undermine people’s intention to receive the COVID-19 vaccine in Austria? *Journal of Community Psychology*, *50*(3), 1269–1281. https://doi.org/10.1002/jcop.22714
135. Kohút, M., Šrol, J., & Čavojová, V. (2022). How are you holding up? Personality, cognitive and social predictors of a perceived shift in subjective well-being during COVID-19 pandemic. *Personality and Individual Differences*, *186*, 111349. https://doi.org/10.1016/j.paid.2021.111349
136. Kosarkova, A., Malinakova, K., van Dijk, J. P., & Tavel, P. (2021). Vaccine Refusal in the Czech Republic Is Associated with Being Spiritual but Not Religiously Affiliated. *Vaccines*, *9*(10), 1157. https://doi.org/10.3390/vaccines9101157
137. Kovic, M., & Füchslin, T. (2018). Probability and conspiratorial thinking. *Applied Cognitive Psychology*, *32*(3), 390–400. https://doi.org/10.1002/acp.3408
138. Kowalski, J., Marchlewska, M., Molenda, Z., Górska, P., & Gawęda, Ł. (2020). Adherence to safety and self-isolation guidelines, conspiracy and paranoia-like beliefs during COVID-19 pandemic in Poland—Associations and moderators. *Psychiatry Research*, *294*, 113540. https://doi.org/10.1016/j.psychres.2020.113540
139. Krüppel, J., Yoon, D., Fieg, K., Sharma, P., & Mokros, A. (2021). On the relationship between right-wing attitudes, conspiracy beliefs, and intergroup threat: Introducing an indirect measure for intergroup threat. *Journal of Theoretical Social Psychology*, *5*(4), 354–365. https://doi.org/10.1002/jts5.103
140. Kuhn, S. A. K., Lieb, R., Freeman, D., Andreou, C., & Zander-Schellenberg, T. (2021). Coronavirus conspiracy beliefs in the German-speaking general population: Endorsement rates and links to reasoning biases and paranoia. *Psychological Medicine*, 1–15. https://doi.org/10.1017/S0033291721001124
141. Lamberty, P., & Imhoff, R. (2018). Powerful Pharma and Its Marginalized Alternatives? *Social Psychology*, *49*(5), 255–270. https://doi.org/10.1027/1864-9335/a000347
142. Lamberty, P. K., Hellmann, J. H., & Oeberst, A. (2018). The winner knew it all? Conspiracy beliefs and hindsight perspective after the 2016 US general election. *Personality and Individual Differences*, *123*, 236–240.
143. Landrum, A. R., & Olshansky, A. (2019). The role of conspiracy mentality in denial of science and susceptibility to viral deception about science. *Politics and the Life Sciences*, *38*(2), 193–209. https://doi.org/10.1017/pls.2019.9
144. Landrum, A. R., Olshansky, A., & Richards, O. (2021). Differential susceptibility to misleading flat earth arguments on youtube. *Media Psychology*, *24*(1), 136–165. https://doi.org/10.1080/15213269.2019.1669461
145. Lantian, A., Bagneux, V., Delouvée, S., & Gauvrit, N. (2021). Maybe a free thinker but not a critical one: High conspiracy belief is associated with low critical thinking ability. *Applied Cognitive Psychology*, *35*(3), 674–684.
146. Lantian, A., Muller, D., Nurra, C., Klein, O., Berjot, S., & Pantazi, M. (2018). Stigmatized beliefs: Conspiracy theories, anticipated negative evaluation of the self, and fear of social exclusion. *European Journal of Social Psychology*, *48*(7), 939–954. https://doi.org/10.1002/ejsp.2498
147. Larsen, E. M., Donaldson, K. R., Liew, M., & Mohanty, A. (2021). Conspiratorial Thinking During COVID-19: The Roles of Paranoia, Delusion-Proneness, and Intolerance of Uncertainty. *Frontiers in Psychiatry*, *12*, 698147. https://doi.org/10.3389/fpsyt.2021.698147
148. Latkin, C. A., Dayton, L., Moran, M., Strickland, J. C., & Collins, K. (2021). Behavioral and psychosocial factors associated with COVID-19 skepticism in the United States. *Current Psychology*, 1-9.
149. Lazarević, L. B., Purić, D., Teovanović, P., Lukić, P., Zupan, Z., & Knežević, G. (2021). What drives us to be (ir)responsible for our health during the COVID-19 pandemic? The role of personality, thinking styles, and conspiracy mentality. *Personality and Individual Differences*, *176*, 110771. https://doi.org/10.1016/j.paid.2021.110771
150. Leibovitz, T., Shamblaw, A. L., Rumas, R., & Best, M. W. (2021). COVID-19 conspiracy beliefs: Relations with anxiety, quality of life, and schemas. *Personality and Individual Differences*, *175*, 110704.
151. Leone, L., Giacomantonio, M., & Lauriola, M. (2019). Moral foundations, worldviews, moral absolutism and belief in conspiracy theories. *International Journal of Psychology*, *54*(2), 197–204. https://doi.org/10.1002/ijop.12459
152. Leone, L., Giacomantonio, M., Williams, R., & Michetti, D. (2018). Avoidant attachment style and conspiracy ideation. *Personality and Individual Differences*, *134*, 329–336. https://doi.org/10.1016/j.paid.2018.06.043
153. Lindholt, M. F., Jørgensen, F., Bor, A., & Petersen, M. B. (2021). Public acceptance of COVID-19 vaccines: Cross-national evidence on levels and individual-level predictors using observational data. *BMJ Open*, *11*(6), e048172. https://doi.org/10.1136/bmjopen-2020-048172
154. Lobato, E. J. C., Powell, M., Padilla, L. M. K., & Holbrook, C. (2020). Factors Predicting Willingness to Share COVID-19 Misinformation. *Frontiers in Psychology*, *11*. https://www.frontiersin.org/article/10.3389/fpsyg.2020.566108
155. Lyons, B., Merola, V., & Reifler, J. (2019). Not Just Asking Questions: Effects of Implicit and Explicit Conspiracy Information About Vaccines and Genetic Modification. *Health Communication*, *34*(14), 1741–1750. https://doi.org/10.1080/10410236.2018.1530526
156. Maftei, A., & Holman, A. C. (2021). SARS-CoV-2 Threat Perception and Willingness to Vaccinate: The Mediating Role of Conspiracy Beliefs. *Frontiers in Psychology*, *12*. https://www.frontiersin.org/article/10.3389/fpsyg.2021.672634
157. Maftei, A., & Holman, A.-C. (2022). Beliefs in conspiracy theories, intolerance of uncertainty, and moral disengagement during the coronavirus crisis. *Ethics & Behavior*, *32*(1), 1–11. https://doi.org/10.1080/10508422.2020.1843171
158. Maglić, M., Pavlović, T., & Franc, R. (2021). Analytic Thinking and Political Orientation in the Corona Crisis. *Frontiers in Psychology*, *12*. https://www.frontiersin.org/article/10.3389/fpsyg.2021.631800
159. Majima, Y., & Nakamura, H. (2020). Development of the Japanese Version of the Generic Conspiracist Beliefs Scale (GCBS‐J). *Japanese Psychological Research*, 62(4), 254-267.
160. Mancosu, M., Ladini, R., & Vassallo, S. (2021). Political consequences of conspiratorial thinking: Evidence from 2016 Italian constitutional referendum. *Acta Politica*, *56*(1), 69–88. https://doi.org/10.1057/s41269-019-00146-3
161. Mao, J.-Y., Yang, S.-L., & Guo, Y.-Y. (2020). Are individuals from lower social classes more susceptible to conspiracy theories? An explanation from the compensatory control theory. *Asian Journal of Social Psychology*, *23*(4), 372–383. https://doi.org/10.1111/ajsp.12417
162. March, E., & Springer, J. (2019). Belief in conspiracy theories: The predictive role of schizotypy, Machiavellianism, and primary psychopathy. *PLOS ONE*, *14*(12), e0225964. https://doi.org/10.1371/journal.pone.0225964
163. Marchlewska, M., Cichocka, A., & Kossowska, M. (2018). Addicted to answers: Need for cognitive closure and the endorsement of conspiracy beliefs. *European Journal of Social Psychology*, *48*(2), 109–117.
164. Marchlewska, M., Cichocka, A., \Lozowski, F., Górska, P., & Winiewski, M. (2019). In search of an imaginary enemy: Catholic collective narcissism and the endorsement of gender conspiracy beliefs. *The Journal of Social Psychology*, *159*(6), 766–779.
165. Marchlewska, M., Green, R., Cichocka, A., Molenda, Z., & Douglas, K. M. (2021). From bad to worse: Avoidance coping with stress increases conspiracy beliefs. *British Journal of Social Psychology*.
166. Mari, S., Gil de Zúñiga, H., Suerdem, A., Hanke, K., Brown, G., Vilar, R., ... & Bilewicz, M. (2022). Conspiracy theories and institutional trust: examining the role of uncertainty avoidance and active social media use. *Political Psychology*, *43*(2), 277-296.
167. Marinthe, G., Brown, G., Delouvée, S., & Jolley, D. (2020). Looking out for myself: Exploring the relationship between conspiracy mentality, perceived personal risk, and COVID-19 prevention measures. *British Journal of Health Psychology*, *25*(4), 957–980. https://doi.org/10.1111/bjhp.12449
168. Markowitz, D. M., Shoots-Reinhard, B., Peters, E., Silverstein, M. C., Goodwin, R., & Bjälkebring, P. (2021). Dehumanization during the COVID-19 pandemic. *Frontiers in Psychology*, 12, 285.
169. Marques, M. D., Kerr, J. R., Williams, M. N., Ling, M., & McLennan, J. (2021). Associations between conspiracism and the rejection of scientific innovations. *Public Understanding of Science*, *30*(7), 854–867. https://doi.org/10.1177/09636625211007013
170. Marques, M. D., Ling, M., Williams, M. N., Kerr, J. R., & McLennan, J. (2022). Australasian public awareness and belief in conspiracy theories: Motivational correlates. *Political Psychology*, *43*(1), 177–198. https://doi.org/10.1111/pops..12746
171. Martinez-Berman, L., McCutcheon, L., & Huynh, H. P. (2021). Is the worship of celebrities associated with resistance to vaccinations? Relationships between celebrity admiration, anti-vaccination attitudes, and beliefs in conspiracy. *Psychology, Health & Medicine*, *26*(9), 1063–1072. https://doi.org/10.1080/13548506.2020.1778754
172. Mashuri, A., van Leeuwen, E., Zaduqisti, E., Sukmawati, F., Sakdiah, H., & Herani, I. (2022). The psychological antecedents of resistance to humanitarian aid. *Group Processes & Intergroup Relations*, *25*(1), 280–297. https://doi.org/10.1177/1368430220962179
173. Meuer, M., & Imhoff, R. (2021). Believing in hidden plots is associated with decreased behavioral trust: Conspiracy belief as greater sensitivity to social threat or insensitivity towards its absence? *Journal of Experimental Social Psychology*, *93*, 104081.
174. Meuer, M., Oeberst, A., & Imhoff, R. (2021). Believe It or Not – No Support for an Effect of Providing Explanatory or Threat-Related Information on Conspiracy Theories’ Credibility. *International Review of Social Psychology*, *34*(1), 26. https://doi.org/10.5334/irsp.587
175. Mikušková, E. (2021). The Analytic Cognitive Style and Conspiracy Mentality as Predictors of Conspiracy Beliefs. *Studia Psychologica*, *63*, 190–203. https://doi.org/10.31577/sp.2021.02.819
176. Miller, J. M. (2020). Do COVID-19 conspiracy theory beliefs form a monological belief system? *Canadian Journal of Political Science/Revue Canadienne de Science Politique*, *53*(2), 319–326.
177. Milošević Đorđević, J., Mari, S., Vdović, M., & Milošević, A. (2021). Links between conspiracy beliefs, vaccine knowledge, and trust: Anti-vaccine behavior of Serbian adults. *Social Science & Medicine*, *277*, 113930. https://doi.org/10.1016/j.socscimed.2021.113930
178. Milošević Đorđević, J., Žeželj, I., & Đurić, Ž. (2021). Beyond General Political Attitudes: Conspiracy Mentality as a Global Belief System Predicts Endorsement of International and Local Conspiracy Theories. *Journal of Social and Political Psychology*, *9*(1), 144–158. https://doi.org/10.5964/jspp.5609
179. Min, S. J. (2021). Who Believes in Conspiracy Theories? Network Diversity, Political Discussion, and Conservative Conspiracy Theories on Social Media. *American Politics Research*, *49*(5), 415–427. https://doi.org/10.1177/1532673X211013526
180. Molz, G., & Stiller, M. (2021). Attitudes and opinions about refugees in germany – correlates with conspiracy and political mindsets. *Current Psychology*, *40*(5), 2201–2210. https://doi.org/10.1007/s12144-018-0108-0
181. Mondak, J. J. (2020). Citizen grit: Effects of domain-specificity, perseverance, and consistency on political judgment. *Personality and Individual Differences*, *163*, 110059.
182. Natoli, E. E., & Marques, M. D. (2021). The antidepressant hoax: Conspiracy theories decrease health-seeking intentions. *British Journal of Social Psychology*, *60*(3), 902–923. https://doi.org/10.1111/bjso.12426
183. Naveed, M. A., Malik, A., & Mahmood, K. (2021). Impact of conspiracy beliefs on Covid-19 fear and health protective behavior: A case of university students. *Library Hi Tech*, *39*(3), 761–775. https://doi.org/10.1108/LHT-12-2020-0322
184. Nazlı, Ş. B., Yığman, F., Sevindik, M., & Deniz Özturan, D. (2022). Psychological factors affecting COVID-19 vaccine hesitancy. *Irish Journal of Medical Science (1971 -)*, *191*(1), 71–80. https://doi.org/10.1007/s11845-021-02640-0
185. Nera, K., Pantazi, M., & Klein, O. (2018). “These Are Just Stories, Mulder”: Exposure to Conspiracist Fiction Does Not Produce Narrative Persuasion. *Frontiers in Psychology*, *9*. https://www.frontiersin.org/article/10.3389/fpsyg.2018.00684
186. Nera, K., Wagner-Egger, P., Bertin, P., Douglas, K. M., & Klein, O. (2021). A power-challenging theory of society, or a conservative mindset? Upward and downward conspiracy theories as ideologically distinct beliefs. *European Journal of Social Psychology*, *51*(4–5), 740–757. https://doi.org/10.1002/ejsp.2769
187. Nestik, T. A., Deyneka, O. S., & Maksimenko, A. А. (2021). Socio-psychological predictors of belief in conspiracy theories of the origin of COVID-19 and involvement in social media. *Social Psychology and Society*, *11*(4), 87–104. Scopus. https://doi.org/10.17759/SPS.2020110407
188. Nyhan, B., & Zeitzoff, T. (2018). Conspiracy and Misperception Belief in the Middle East and North Africa. *The Journal of Politics*, *80*(4), 1400–1404. https://doi.org/10.1086/698663
189. Ojikutu, B. O., Amutah-Onukagha, N., Mahoney, T. F., Tibbitt, C., Dale, S. D., Mayer, K. H., & Bogart, L. M. (2020). HIV-Related Mistrust (or HIV Conspiracy Theories) and Willingness to Use PrEP Among Black Women in the United States. *AIDS and Behavior*, *24*(10), 2927–2934. https://doi.org/10.1007/s10461-020-02843-z
190. Olansky, E., Mansergh, G., Pitts, N., Mimiaga, M. J., Denson, D. J., Landers, S., Holman, J., & Herbst, J. H. (2020). PrEP Awareness in the Context of HIV/AIDS Conspiracy Beliefs Among Black/African American and Hispanic/Latino MSM in Three Urban US Cities. *Journal of Homosexuality*, *67*(6), 833–843. https://doi.org/10.1080/00918369.2018.1557953
191. Oleksy, T., Wnuk, A., Gambin, M., & Łyś, A. (2021a). Dynamic relationships between different types of conspiracy theories about COVID-19 and protective behaviour: A four-wave panel study in Poland. *Social Science & Medicine*, *280*, 114028. https://doi.org/10.1016/j.socscimed.2021.114028
192. Oleksy, T., Wnuk, A., Maison, D., & Łyś, A. (2021b). Content matters. Different predictors and social consequences of general and government-related conspiracy theories on COVID-19. *Personality and Individual Differences*, *168*, 110289. https://doi.org/10.1016/j.paid.2020.110289
193. Onderco, M., & Stoeckel, F. (2020). Conspiratorial thinking and foreign policy views: Evidence from Central Europe. *Journal of Elections, Public Opinion and Parties*, *0*(0), 1–15. https://doi.org/10.1080/17457289.2020.1814309
194. Pantazi, M., Papaioannou, K., & van Prooijen, J.-W. (2021). Power to the People: The Hidden Link Between Support for Direct Democracy and Belief in Conspiracy Theories. *Political Psychology*, *43*(3), 529–548. https://doi.org/10.1111/pops.12779
195. Parent, M. C., Woznicki, N., Dillon, F. R., & Pituch, K. A. (2020). Psychosocial barriers to pre-exposure prophylaxis (PrEP) uptake: The roles of heterosexual self-presentation, sexual risk, and perceived peer prep use. *Psychology of Men & Masculinities*, *21*(4), 699–703. https://doi.org/10.1037/men0000258
196. Patev, A. J., Hood, K. B., Speed, K. J., Cartwright, P. M., & Kinman, B. A. (2019). HIV conspiracy theory beliefs mediates the connection between HIV testing attitudes and HIV prevention self-efficacy. *Journal of American College Health*, *67*(7), 661–673. https://doi.org/10.1080/07448481.2018.1500472
197. Pavela Banai, I., Banai, B., & Mikloušić, I. (2021). Beliefs in COVID-19 conspiracy theories, compliance with the preventive measures, and trust in government medical officials. *Current Psychology*. https://doi.org/10.1007/s12144-021-01898-y
198. Pellegrini, V., Giacomantonio, M., De Cristofaro, V., Salvati, M., Brasini, M., Carlo, E., Mancini, F., & Leone, L. (2021). Is Covid-19 a natural event? Covid-19 pandemic and conspiracy beliefs. *Personality and Individual Differences*, *181*, 111011.
199. Pennycook, G., Cheyne, J. A., Koehler, D. J., & Fugelsang, J. A. (2020). On the belief that beliefs should change according to evidence: Implications for conspiratorial, moral, paranormal, political, religious, and science beliefs. *Judgment and Decision Making*, *15*(4), 476.
200. Petelinšek, A., & Lauri Korajlija, A. (2020). Predictors of pharmacophobia. *Health Psychology Research*, *8*(1), 8853. https://doi.org/10.4081/hpr.2020.8853
201. Petersen, M. B., Bor, A., Jørgensen, F., & Lindholt, M. F. (2021). Transparent communication about negative features of COVID-19 vaccines decreases acceptance but increases trust. *Proceedings of the National Academy of Sciences*, *118*(29), e2024597118. https://doi.org/10.1073/pnas.2024597118
202. Petrović, B., Međedović, J., Radović, O., & Radetić Lovrić, S. (2019). Conspiracy Mentality in Post-Conflict Societies: Relations With the Ethos of Conflict and Readiness for Reconciliation. *Europe’s Journal of Psychology*, *15*(1), 59–81. https://doi.org/10.5964/ejop.v15i1.1695
203. Petrović, M. B., & Žeželj, I. (2021). Thinking inconsistently: Development and validation of an instrument for assessing proneness to doublethink. *European Journal of Psychological Assessment*, No Pagination Specified-No Pagination Specified. https://doi.org/10.1027/1015-5759/a000645
204. Piltch-Loeb, R., Zikmund-Fisher, B. J., Shaffer, V. A., Scherer, L. D., Knaus, M., Fagerlin, A., Abramson, D. M., & Scherer, A. M. (2019). Cross-Sectional Psychological and Demographic Associations of Zika Knowledge and Conspiracy Beliefs Before and After Local Zika Transmission. *Risk Analysis*, *39*(12), 2683–2693. https://doi.org/10.1111/risa.13369
205. Pisl, V., Volavka, J., Chvojkova, E., Cechova, K., Kavalirova, G., & Vevera, J. (2021a). Dissociation, Cognitive Reflection and Health Literacy Have a Modest Effect on Belief in Conspiracy Theories about COVID-19. *International Journal of Environmental Research and Public Health*, *18*(10), 5065. https://doi.org/10.3390/ijerph18105065
206. Pisl, V., Volavka, J., Chvojkova, E., Cechova, K., Kavalirova, G., & Vevera, J. (2021b). Willingness to Vaccinate Against COVID-19: The Role of Health Locus of Control and Conspiracy Theories. *Frontiers in Psychology*, *12*, 717960. https://doi.org/10.3389/fpsyg.2021.717960
207. Pivetti, M., Di Battista, S., Paleari, F. G., & Hakoköngäs, E. (2021a). Conspiracy beliefs and attitudes toward COVID-19 vaccinations: A conceptual replication study in Finland. *Journal of Pacific Rim Psychology*, *15*, 18344909211039892. https://doi.org/10.1177/18344909211039893
208. Pivetti, M., Melotti, G., Bonomo, M., & Hakoköngäs, E. (2021b). Conspiracy Beliefs and Acceptance of COVID-Vaccine: An Exploratory Study in Italy. *Social Sciences*, *10*(3), 108. https://doi.org/10.3390/socsci10030108
209. Plohl, N., & Musil, B. (2021). Modeling compliance with COVID-19 prevention guidelines: The critical role of trust in science. *Psychology, Health & Medicine*, *26*(1), 1–12. https://doi.org/10.1080/13548506.2020.1772988
210. Poon, K.-T., Chen, Z., & Wong, W.-Y. (2020). Beliefs in conspiracy theories following ostracism. *Personality and Social Psychology Bulletin*, *46*(8), 1234–1246.
211. Prichard, E. C., & Christman, S. D. (2020). Authoritarianism, Conspiracy Beliefs, Gender and COVID-19: Links Between Individual Differences and Concern About COVID-19, Mask Wearing Behaviors, and the Tendency to Blame China for the Virus. *Frontiers in Psychology*, *11*. https://www.frontiersin.org/article/10.3389/fpsyg.2020.597671
212. Pummerer, L., Böhm, R., Lilleholt, L., Winter, K., Zettler, I., & Sassenberg, K. (2022). Conspiracy Theories and Their Societal Effects During the COVID-19 Pandemic. *Social Psychological and Personality Science*, *13*(1), 49–59. https://doi.org/10.1177/19485506211000217
213. Pytlik, N., Soll, D., & Mehl, S. (2020). Thinking Preferences and Conspiracy Belief: Intuitive Thinking and the Jumping to Conclusions-Bias as a Basis for the Belief in Conspiracy Theories. *Frontiers in Psychiatry*, *11*. https://www.frontiersin.org/article/10.3389/fpsyt.2020.568942
214. Radnitz, S. (2021). Dilemmas of Distrust: Conspiracy Beliefs, Elite Rhetoric, and Motivated Reasoning. *Political Research Quarterly*, 10659129211034558. https://doi.org/10.1177/10659129211034558
215. Resnicow, K., Bacon, E., Yang, P., Hawley, S., Horn, M. L. V., & An, L. (2021). Novel Predictors of COVID-19 Protective Behaviors Among US Adults: Cross-sectional Survey. *Journal of Medical Internet Research*, *23*(4), e23488. https://doi.org/10.2196/23488
216. Rezende, A. T., Gouveia, V. V., Nascimento, A. M. do, Vilar, R., & Oliveira, K. G. (2019). Correlatos valorativos de creencias en teorías de conspiración (Values Correlates of Beliefs in Conspiracy Theories). *Avances En Psicología Latinoamericana*, *37*(2), 219–234. https://doi.org/10.12804/revistas.urosario.edu.co/apl/a.7211
217. Rezende, A. T., Gouveia, V. V., Soares, A. K. S., Moizéis, H. B. C., Gouveia, V. V., Soares, A. K. S., & Moizéis, H. B. C. (2021). Creencias de las teorías de la conspiración en estudiantes universitarios: Una explicación basada en los rasgos de personalidad (Beliefs in Conspiracy Theories in University Students: An Explanation Based on Personality Traits. *Psicología, Conocimiento y Sociedad*, *11*(2), 84–98. https://doi.org/10.26864/pcs.v11.n2.6
218. Rieger, M. O. (2020). What Makes Young People Think Positively About Social Distancing During the Corona Crisis in Germany? *Frontiers in Sociology*, *5*. https://www.frontiersin.org/article/10.3389/fsoc.2020.00061
219. Rizeq, J., Flora, D. B., & Toplak, M. E. (2021). An examination of the underlying dimensional structure of three domains of contaminated mindware: paranormal beliefs, conspiracy beliefs, and anti-science attitudes. T*hinking & Reasoning,* 27(2), 187-211.
220. Romer, D., & Jamieson, K. H. (2020). Conspiracy theories as barriers to controlling the spread of COVID-19 in the U.S. *Social Science & Medicine*, *263*, 113356. https://doi.org/10.1016/j.socscimed.2020.113356
221. Rottweiler, B., & Gill, P. (2020). Conspiracy Beliefs and Violent Extremist Intentions: The Contingent Effects of Self-efficacy, Self-control and Law-related Morality. *Terrorism and Political Violence*, *0*(0), 1–20. https://doi.org/10.1080/09546553.2020.1803288
222. Rozbroj, T., Lyons, A., & Lucke, J. (2019). Psychosocial and demographic characteristics relating to vaccine attitudes in Australia. *Patient Education and Counseling*, *102*(1), 172–179. https://doi.org/10.1016/j.pec.2018.08.027
223. Ruiz, J. B., & Bell, R. A. (2021). Predictors of intention to vaccinate against COVID-19: Results of a nationwide survey. *Vaccine*, *39*(7), 1080–1086. https://doi.org/10.1016/j.vaccine.2021.01.010
224. Sallam, M., Dababseh, D., Eid, H., Al-Mahzoum, K., Al-Haidar, A., Taim, D., Yaseen, A., Ababneh, N. A., Bakri, F. G., & Mahafzah, A. (2021a). High Rates of COVID-19 Vaccine Hesitancy and Its Association with Conspiracy Beliefs: A Study in Jordan and Kuwait among Other Arab Countries. *Vaccines*, *9*(1), 42. https://doi.org/10.3390/vaccines9010042
225. Sallam, M., Dababseh, D., Eid, H., Hasan, H., Taim, D., Al-Mahzoum, K., Al-Haidar, A., Yaseen, A., Ababneh, N. A., Assaf, A., Bakri, F. G., Matar, S., & Mahafzah, A. (2021b). Low COVID-19 Vaccine Acceptance Is Correlated with Conspiracy Beliefs among University Students in Jordan. *International Journal of Environmental Research and Public Health*, *18*(5), 2407. https://doi.org/10.3390/ijerph18052407
226. Sallam, M., Dababseh, D., Yaseen, A., Al-Haidar, A., Ababneh, N. A., Bakri, F. G., & Mahafzah, A. (2020). Conspiracy Beliefs Are Associated with Lower Knowledge and Higher Anxiety Levels Regarding COVID-19 among Students at the University of Jordan. *International Journal of Environmental Research and Public Health*, *17*(14), 4915. https://doi.org/10.3390/ijerph17144915
227. Sanchez, C., & Dunning, D. (2021). Jumping to conclusions: Implications for reasoning errors, false belief, knowledge corruption, and impeded learning. *Journal of Personality and Social Psychology*, *120*(3), 789–815. https://doi.org/10.1037/pspp0000375
228. Schlipphak, B., Bollwerk, M., & Back, M. (2021). Beliefs in conspiracy theories (CT): The role of country context. *Political Research Exchange*, *3*(1), 1949358. https://doi.org/10.1080/2474736X.2021.1949358
229. Schnell, T., Spitzenstätter, D., & Krampe, H. (2021). Compliance with COVID-19 public health guidelines: An attitude-behaviour gap bridged by personal concern and distance to conspiracy ideation. *Psychology & Health*, *0*(0), 1–22. https://doi.org/10.1080/08870446.2021.1974861
230. Schnepf, J., Lux, A., Jin, Z., & Formanowicz, M. (2021). Left Out—Feelings of Social Exclusion Incite Individuals with High Conspiracy Mentality to Reject Complex Scientific Messages. *Journal of Language and Social Psychology*, *40*(5–6), 627–652. https://doi.org/10.1177/0261927X211044789
231. Scrima, F., Miceli, S., Caci, B., & Cardaci, M. (2022). The relationship between fear of COVID-19 and intention to get vaccinated. The serial mediation roles of existential anxiety and conspiracy beliefs. *Personality and Individual Differences*, *184*, 111188. https://doi.org/10.1016/j.paid.2021.111188
232. Siddiqui, N. (2020). Who do you believe? Political parties and conspiracy theories in Pakistan. *Party Politics*, *26*(2), 107–119. https://doi.org/10.1177/1354068817749777
233. Siem, B., Kretzmeyer, B., & Stürmer, S. (2021). The role of self-evaluation in predicting attitudes toward supporters of COVID-19-related conspiracy theories: A direct and a conceptual replication of Cichocka et al.(2016). *Journal of Pacific Rim Psychology*, *15*, 18344909211052588.
234. Simione, L., Vagni, M., Gnagnarella, C., Bersani, G., & Pajardi, D. (2021). Mistrust and beliefs in conspiracy theories differently mediate the effects of psychological factors on propensity for COVID-19 vaccine. *Frontiers in Psychology*, *12*.
235. Soveri, A., Karlsson, L. C., Antfolk, J., Lindfelt, M., & Lewandowsky, S. (2021). Unwillingness to engage in behaviors that protect against COVID-19: The role of conspiracy beliefs, trust, and endorsement of complementary and alternative medicine. *BMC Public Health*, *21*(1), 684. https://doi.org/10.1186/s12889-021-10643-w
236. Sowa, P., Kiszkiel, Ł., Laskowski, P. P., Alimowski, M., Szczerbiński, Ł., Paniczko, M., Moniuszko-Malinowska, A., & Kamiński, K. (2021). COVID-19 Vaccine Hesitancy in Poland—Multifactorial Impact Trajectories. *Vaccines*, *9*(8), 876. https://doi.org/10.3390/vaccines9080876
237. Spasovski, O., & Kenig, K. (2020). PSYCHOLOGICAL WELL-BEING IN STUDENTS DURING SELF-ISOLATION DUE TO THE COVID-19 PANDEMIC. *Primenjena Psihologija*, *13*(4), 427–477.
238. Šrol, J., Ballová Mikušková, E., & Čavojová, V. (2021). When we are worried, what are we thinking? Anxiety, lack of control, and conspiracy beliefs amidst the COVID-19 pandemic. *Applied Cognitive Psychology*, *35*(3), 720–729. https://doi.org/10.1002/acp.3798
239. Ståhl, T., & van Prooijen, J.-W. (2018). Epistemic rationality: Skepticism toward unfounded beliefs requires sufficient cognitive ability and motivation to be rational. *Personality and Individual Differences*, *122*, 155–163. https://doi.org/10.1016/j.paid.2017.10.026
240. Stecula, D. A., & Pickup, M. (2021). How populism and conservative media fuel conspiracy beliefs about COVID-19 and what it means for COVID-19 behaviors. *Research & Politics*, *8*(1), 2053168021993979. https://doi.org/10.1177/2053168021993979
241. Stoica, C. A., & Umbreș, R. (2021). Suspicious minds in times of crisis: Determinants of Romanians’ beliefs in COVID-19 conspiracy theories. *European Societies*, *23*(sup1), S246–S261. https://doi.org/10.1080/14616696.2020.1823450
242. Stojanov, A., & Douglas, K. (2022). Conspiracy beliefs in Britain and North Macedonia: A comparative study. *International Journal of Psychology*, *57*(2), 209–217. https://doi.org/10.1002/ijop.12801
243. Stojanov, A., & Halberstadt, J. (2019). The Conspiracy Mentality Scale: Distinguishing between irrational and rational suspicion. *Social Psychology*, 50(4), 215.
244. Stojanov, J., Stanisavljavić, S., Tatić, V., & Pantić, A. (2019). Conspiracy Thinking Inventory (Cti)–Construction And Validation Study. *Primenjena Psihologija*, 12(4), 363-383.
245. Stojanov, A., Halberstadt, J., Bering, J. M., & Kenig, N. (2021). Examining a domain-specific link between perceived control and conspiracy beliefs: A brief report in the context of COVID-19. *Current Psychology*. https://doi.org/10.1007/s12144-021-01977-0
246. Šuriņa, S., Martinsone, K., Perepjolkina, V., Kolesnikova, J., Vainik, U., Ruža, A., Vrublevska, J., Smirnova, D., Fountoulakis, K. N., & Rancans, E. (2021). Factors Related to COVID-19 Preventive Behaviors: A Structural Equation Model. *Frontiers in Psychology*, *12*, 676521. https://doi.org/10.3389/fpsyg.2021.676521
247. Swami, V., & Barron, D. (2021). Rational thinking style, rejection of coronavirus (COVID-19) conspiracy theories/theorists, and compliance with mandated requirements: Direct and indirect relationships in a nationally representative sample of adults from the United Kingdom. *Journal of Pacific Rim Psychology*, *15*, 18344909211037384. https://doi.org/10.1177/18344909211037385
248. Swami, V., Barron, D., Weis, L., & Furnham, A. (2018). To Brexit or not to Brexit: The roles of Islamophobia, conspiracist beliefs, and integrated threat in voting intentions for the United Kingdom European Union membership referendum. *British Journal of Psychology*, *109*(1), 156–179. https://doi.org/10.1111/bjop.12252
249. Teličák, P., & Halama, P. (2021). Maladaptive Personality Traits, Religiosity and Spirituality as Predictors of Epistemically Unfounded Beliefs. *Studia Psychologica*, *63*(2), 175–189.
250. Teovanović, P., Lukić, P., Zupan, Z., Lazić, A., Ninković, M., & Žeželj, I. (2021). Irrational beliefs differentially predict adherence to guidelines and pseudoscientific practices during the COVID-19 pandemic. *Applied Cognitive Psychology*, *35*(2), 486–496. https://doi.org/10.1002/acp.3770
251. Tonković, M., Dumančić, F., Jelić, M., & Biruški, D. Č. (2021). Who believes in COVID-19 conspiracy theories in Croatia? Prevalence and predictors of conspiracy beliefs. *Frontiers in Psychology*, *12*.
252. van der Linden, S., Panagopoulos, C., Azevedo, F., & Jost, J. T. (2021). The Paranoid Style in American Politics Revisited: An Ideological Asymmetry in Conspiratorial Thinking. *Political Psychology*, *42*(1), 23–51. https://doi.org/10.1111/pops.12681
253. van der Wal, R. C., Sutton, R. M., Lange, J., & Braga, J. P. N. (2018). Suspicious binds: Conspiracy thinking and tenuous perceptions of causal connections between co-occurring and spuriously correlated events. *European Journal of Social Psychology*, *48*(7), 970–989. https://doi.org/10.1002/ejsp.2507
254. van Prooijen, J.-W., Douglas, K. M., & De Inocencio, C. (2018). Connecting the dots: Illusory pattern perception predicts belief in conspiracies and the supernatural. *European Journal of Social Psychology*, *48*(3), 320–335. https://doi.org/10.1002/ejsp.2331
255. van Prooijen, J.-W., Etienne, T. W., Kutiyski, Y., & Krouwel, A. P. (2021a). Just a Flu? Self-perceived infection mediates the link between conspiracy beliefs and Covid-19 health beliefs and behaviors. *Journal of Health Psychology*, 13591053211051816. https://doi.org/10.1177/13591053211051816
256. van Prooijen, J.-W., Ligthart, J., Rosema, S., & Xu, Y. (2022). The entertainment value of conspiracy theories. *British Journal of Psychology*, *113*(1), 25–48. https://doi.org/10.1111/bjop.12522
257. van Prooijen, J.-W., & Song, M. (2021). The cultural dimension of intergroup conspiracy theories. *British Journal of Psychology*, *112*(2), 455–473. https://doi.org/10.1111/bjop.12471
258. van Prooijen, J.-W., Staman, J., & Krouwel, A. P. M. (2018). Increased conspiracy beliefs among ethnic and Muslim minorities. *Applied Cognitive Psychology*, *32*(5), 661–667. https://doi.org/10.1002/acp.3442
259. van Prooijen, J.-W. van, Etienne, T. W., Kutiyski, Y., & Krouwel, A. P. M. (2021b). Conspiracy beliefs prospectively predict health behavior and well-being during a pandemic. *Psychological Medicine*, 1–8. https://doi.org/10.1017/S0033291721004438
260. Vezzoni, C., Sani, G. M. D., Chiesi, A. M., Ladini, R., Biolcati, F., Guglielmi, S., Maggini, N., Maraffi, M., Molteni, F., Pedrazzani, A., & Segatti, P. (2022). Where does the Coronavirus come from? On the mechanisms underlying the endorsement of conspiracy theories on the origin of SARS-CoV-2. *Italian Political Science Review / Rivista Italiana Di Scienza Politica*, *52*(1), 51–65. https://doi.org/10.1017/ipo.2021.19
261. Vitriol, J. A., & Marsh, J. K. (2018). The illusion of explanatory depth and endorsement of conspiracy beliefs. *European Journal of Social Psychology*, *48*(7), 955–969.
262. Wabnegger, A., Gremsl, A., & Schienle, A. (2021). The association between the belief in coronavirus conspiracy theories, miracles, and the susceptibility to conjunction fallacy. *Applied Cognitive Psychology*, *35*(5), 1344–1348.
263. Walter, A. S., & Drochon, H. (2022). Conspiracy thinking in Europe and America: A comparative study. *Political Studies,* 70(2), 483-501.
264. Wang, J., & Kim, S. (2021). The Paradox of Conspiracy Theory: The Positive Impact of Beliefs in Conspiracy Theories on Preventive Actions and Vaccination Intentions during the COVID-19 Pandemic. *International Journal of Environmental Research and Public Health*, *18*(22), 11825. https://doi.org/10.3390/ijerph182211825
265. Wagner-Egger, P., Delouvée, S., Gauvrit, N., and Dieguez, S. (2018). Creationism and conspiracism share a common teleological bias. Current Biology 28, R867–R868. doi: 10.1016/j.cub.2018.06.072.
266. Wang, X., Zuo, S.-J., Chan, H.-W., Chiu, C. P.-Y., & Hong, Y. (2021). COVID-19-related conspiracy theories in China: The role of secure versus defensive in-group positivity and responsibility attributions. *Journal of Pacific Rim Psychology*, *15*, 18344909211034930. https://doi.org/10.1177/18344909211034928
267. Whitson, J. A., Kim, J., Wang, C. S., Menon, T., & Webster, B. D. (2019). Regulatory Focus and Conspiratorial Perceptions: The Importance of Personal Control. *Personality and Social Psychology Bulletin*, *45*(1), 3–15. https://doi.org/10.1177/0146167218775070
268. Winter, K., Pummerer, L., Hornsey, M. J., & Sassenberg, K. (2022). Pro-vaccination subjective norms moderate the relationship between conspiracy mentality and vaccination intentions. *British Journal of Health Psychology*, *27*(2), 390–405. https://doi.org/10.1111/bjhp.12550
269. Wirawan, G. B. S., Mahardani, P. N. T. Y., Cahyani, M. R. K., Laksmi, N. L. P. S. P., & Januraga, P. P. (2021). Conspiracy beliefs and trust as determinants of COVID-19 vaccine acceptance in Bali, Indonesia: Cross-sectional study. *Personality and Individual Differences*, *180*, 110995. https://doi.org/10.1016/j.paid.2021.110995
270. Wood, M. J., & Gray, D. (2019). Right-wing authoritarianism as a predictor of pro-establishment versus anti-establishment conspiracy theories. *Personality and Individual Differences*, *138*, 163–166.
271. Woolf, K., McManus, I. C., Martin, C. A., Nellums, L. B., Guyatt, A. L., Melbourne, C., Bryant, L., Gogoi, M., Wobi, F., Al-Oraibi, A., Hassan, O., Gupta, A., John, C., Tobin, M. D., Carr, S., Simpson, S., Gregary, B., Aujayeb, A., Zingwe, S., … Pareek, M. (2021). Ethnic differences in SARS-CoV-2 vaccine hesitancy in United Kingdom healthcare workers: Results from the UK-REACH prospective nationwide cohort study. *The Lancet Regional Health - Europe*, *9*, 100180. https://doi.org/10.1016/j.lanepe.2021.100180
272. Yang, Z., Luo, X., & Jia, H. (2021). Is It All a Conspiracy? Conspiracy Theories and People’s Attitude to COVID-19 Vaccination. *Vaccines*, *9*(10), 1051. https://doi.org/10.3390/vaccines9101051
273. Yarosh, N., Artiukhova, V., & Zimovin, O. (2021). Conspiracy Belief and Behavior in the COVID-19 Pandemic. How Belief in Conspiracy Theory Relates to Adherence to Quarantine Restrictions (Wearing Protective Equipment, Isolation, Hygiene) and Influences Antisocial Behavior such as Aggression and Selfishne. *BRAIN. Broad Research in Artificial Intelligence and Neuroscience*, *12*(2), 202–221.
274. Yu, X., Wojcieszak, M., Lee, S., Casas, A., Azrout, R., & Gackowski, T. (2021). The (Null) Effects of Happiness on Affective Polarization, Conspiracy Endorsement, and Deep Fake Recognition: Evidence from Five Survey Experiments in Three Countries. *Political Behavior*, *43*(3), 1265–1287. https://doi.org/10.1007/s11109-021-09701-1
